# Cracking the Blood–Brain Barrier Code: Rational Nanomaterial Design for Next-Generation Neurological Therapies

**DOI:** 10.3390/pharmaceutics17091169

**Published:** 2025-09-06

**Authors:** Lucio Nájera-Maldonado, Mariana Parra-González, Esperanza Peralta-Cuevas, Ashley J. Gutierrez-Onofre, Igor Garcia-Atutxa, Francisca Villanueva-Flores

**Affiliations:** 1 Centro de Investigación en Ciencia Aplicada y Tecnología Avanzada (CICATA) Unidad Morelos del Instituto Politécnico Nacional (IPN), Boulevard de la Tecnología No. 1036, Xochitepec 62790, Mexico; lnajeram2400@alumno.ipn.mx (L.N.-M.); mparrag2400@alumno.ipn.mx (M.P.-G.); mperaltac2400@alumno.ipn.mx (E.P.-C.); ashley.jovannag@gmail.com (A.J.G.-O.); 2 Departamento de Ciencias de la Computación, Universidad Católica de Murcia (UCAM), Av. de los Jerónimos, 135, 30107 Murcia, Spain; igarcia839@alu.ucam.edu

**Keywords:** blood–brain barrier, nanomedicine, CNS drug delivery, nanoparticle, neurotherapeutics

## Abstract

This review provides a mechanistic framework to strategically design nanoparticles capable of efficiently crossing the blood–brain barrier (BBB), a critical limitation in neurological treatments. We systematically analyze nanoparticle–BBB transport mechanisms, including receptor-mediated transcytosis, adsorptive-mediated transcytosis, and transient barrier modulation. Essential nanoparticle parameters (size, shape, stiffness, surface charge, and biofunctionalization) are evaluated for their role in enhancing brain targeting. For instance, receptor-targeted nanoparticles can significantly enhance brain uptake, achieving levels of up to 17.2% injected dose per gram (ID/g) in preclinical glioma models. Additionally, validated preclinical models (human-derived in vitro systems, rodents, and non-human primates) and advanced imaging techniques crucial for assessing nanoparticle performance are discussed. Distinct from prior BBB nanocarrier reviews that primarily catalogue mechanisms, this work (i) derives quantitative ‘design windows’ (size 10–100 nm, aspect ratio ~2–5, near-neutral ζ) linked to transcytosis efficiency, (ii) cross-walks human-relevant in vitro/in vivo models (including TEER thresholds and NHP evidence) into a translational decision guide, and (iii) integrates regulatory/toxicology readiness (ISO 10993-4, FDA/EMA, ICH) into practical checklists. We also curate recent (2020–2025) %ID/g brain-uptake data across lipidic, polymeric, protein, inorganic, and hybrid vectors to provide actionable, evidence-based rules for BBB design.

## 1. Introduction

The blood–brain barrier (BBB) shields the brain from toxins, pathogens, and fluctuations in blood composition through tight junctions between endothelial cells, pericytes, and astrocytic end-feet, forming a highly selective barrier [1]. Its tight junctions (~1.4 nm pore size) and efflux transporters block nearly all large molecules and ~98% of small-molecule drugs [2]. Consequently, systemic therapeutics rarely penetrate effectively; monoclonal antibodies typically achieve <0.1% brain uptake, and even FDA-approved anti-amyloid antibodies for AD reach just ~0.01–0.1% [3]. Similarly, most small-molecule neurotherapeutics require high lipophilicity, yet remain limited by efflux pumps, size, and polarity. Thus, the BBB remains a significant obstacle for CNS drug delivery [1,4].

This barrier-imposed delivery problem leads to several critical challenges in current therapy. First, many efficacious drugs that are effective in vitro or peripheral tissues fail in CNS trials because adequate concentrations in the brain cannot be achieved. For instance, the chemotherapy paclitaxel is potent against glioma cells but is a substrate of P-glycoprotein; when given systemically, it accumulates to only a minute fraction of plasma levels in the brain, rendering it ineffective unless the BBB is modulated [5]. Second, limited BBB permeability forces clinicians to use extreme measures for CNS delivery. High systemic doses are often required to push a small amount of drug across the BBB, which can cause significant off-target toxicity in the rest of the body (e.g., dose-limiting cardiotoxicity of doxorubicin or peripheral immunosuppression by high-dose cytokines). In other cases, drugs must be delivered directly or locally to the CNS, for example, via intrathecal chemotherapy or convection-enhanced delivery in brain tumors, to bypass the BBB. Such invasive approaches are risky, impractical for chronic conditions, and poorly tolerated by patients [6]. Similarly, methods to transiently open the BBB (such as focused ultrasound with microbubbles) can enable higher rates of drug entry but also carry risks of tissue damage or infection, as they allow toxins or pathogens to breach. Owing to these limitations, many CNS diseases remain inadequately treated. In GBM, for instance, standard chemoradiation yields a two-year survival under 20%, in part because drugs like temozolomide, while somewhat BBB-permeable, still do not uniformly reach all tumor cells behind an intact barrier. There is an urgent need for new delivery strategies that can safely and effectively ferry therapeutic molecules across the BBB [7].

To address this, researchers have turned to the rational design of brain-targeted nanocarriers, nanoscale drug delivery systems engineered to traverse the BBB and ferry therapeutic payloads into the brain [8]. This rational design approach focuses on optimizing key physicochemical parameters, namely, nanoparticle size, surface charge, shape, and surface chemistry, because these properties collectively govern BBB permeability and delivery efficiency [9].

Nanotechnology has emerged as a promising avenue to overcome the BBB’s filtering mechanism and improve drug delivery to the brain [10,11,12]. Nanoscale drug carriers, such as polymeric NPs, liposomes, micelles, dendrimers, and protein-based nanovectors, can be engineered to evade or exploit the BBB’s defenses in ways conventional drug formulations cannot. A key advantage is that nanocarriers can be “rationally” designed with specific physicochemical properties (size, shape, surface charge) and functional ligands to actively engage transport pathways of the BBB [9]. In parallel, advances in materials science have led to “smarter” nanobiomaterials that respond to the brain microenvironment. These include stimuli-responsive nanocarriers that release their drug cargo upon sensing pH or enzymatic triggers in the brain, or neutrophil-mimetic NPs that can migrate across an inflamed BBB. These approaches collectively aim to maximize CNS delivery while minimizing systemic exposure [13]. Indeed, targeted nano-delivery can reduce off-target toxicity. In one study, an intranasally administered ferritin NP was shown to concentrate in glioma tissue while largely sparing healthy organs, suggesting a safer profile than intravenous chemotherapy [14,15].

This review provides a robust conceptual and mechanistic framework for rationally designing nanobiotechnological systems capable of effectively crossing the BBB. Rather than merely listing existing delivery methods, we emphasize understanding nanoparticle (NP)–barrier interactions and identifying critical factors underlying successful translocation. We systematically examine key NP transport mechanisms, drawing from recent cellular and in vivo studies, and detail essential physicochemical parameters such as NP size, shape, surface chemistry, targeting ligands, and stimulus-responsive properties. Exemplary systems illustrate successful strategies, including functionalized polymeric NPs, engineered exosomes, and BBB-targeted lipid nanoparticles. Therapeutic applications across various CNS disorders are critically evaluated, showcasing preclinical data demonstrating improved drug delivery, enhanced brain bioavailability, extended survival, and neurological recovery. This review advances beyond earlier surveys of BBB nanocarriers by moving from descriptive mechanism lists to quantitative, design-oriented guidance. First, we consolidate a numerical design space (size, shape, stiffness, ζ-potential) tied to brain uptake and safety. Second, we provide a model-selection map spanning human iPSC BBB chips, rodents, and NHPs with TEER/efflux benchmarks for study design. Third, we include a regulatory/toxicology roadmap (ISO 10993-4 [16]; FDA/EMA; ICH S7A/S8/Q13) with minimum reporting items to accelerate translation. Finally, we synthesize 2020–2025 quantitative %ID/g data and highlight emerging hybrid platforms (exosomes, VLPs, AAV capsids, magnetic guidance) with translational signals not aggregated elsewhere.

## 2. The BBB: Physiology and Restrictive Mechanisms

**Figure 1:**
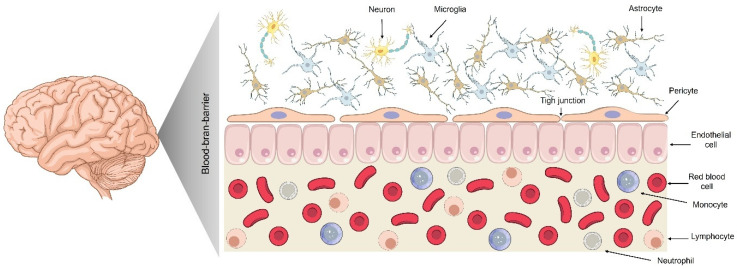
Structural composition of the BBB. The BBB is formed primarily of a monolayer of endothelial cells joined by tight junctions, which restrict paracellular transport. Pericytes and astrocytic end-feet provide both structural and functional support, thereby contributing to the integrity of the barrier and the regulation of nutrient exchange. The neurovascular unit also includes neurons and microglia, which interact closely with vascular components to maintain central nervous system (CNS) homeostasis. Various blood cells circulate in the lumen, including erythrocytes, monocytes, lymphocytes, and neutrophils; however, they are largely excluded from the brain parenchyma under physiological conditions due to the barrier’s selectivity. Modified from [18,19].

A comprehensive understanding of the BBB is fundamental for the rational design of nanocarriers aimed at treating CNS disorders. The BBB is a highly selective physiological interface composed of endothelial cells tightly joined by junctional complexes, supported by pericytes, astrocytic end-feet, and a specialized basement membrane [17] (Figure 1). This intricate structure preserves CNS homeostasis by strictly regulating the passage of molecules between the bloodstream and the brain parenchyma. Its restrictive mechanisms, tight junctions, efflux transporters, and enzymatic barriers pose a significant challenge to drug delivery. Therefore, elucidating the composition, function, and molecular transport mechanisms of the BBB is essential for engineering nanocarriers with optimized properties that can effectively traverse this barrier and deliver therapeutic agents to target sites within the brain.

### 2.1. Composition and Function of the BBB

The BBB’s composition and function comprise brain endothelial cells sealed by tight junctions, supported by pericytes, astrocytic end-feet, and a basement membrane. This neurovascular unit provides selective permeability, maintaining CNS homeostasis [20]. Human brain capillaries extend extensively (~20 m^2^ of surface area), ensuring the proximity of neurons to the blood supply. BBB endothelial cells lack fenestrations and exhibit minimal vesicular transport, restricting transport pathways [21,22,23].

Pericytes, which are abundant in CNS vessels (covering more than 90% of vessels in mice), structurally support the blood–brain barrier and induce endothelial tightness. Astrocytic end-feet regulate BBB function, while the basement membrane (50–100 nm thick) restricts permeability and anchors cells [24,25]. These features yield high transendothelial electrical resistance (TEER) and low permeability, protecting the CNS from blood-derived fluctuations and toxins.

### 2.2. Tight Junctions and Paracellular Restriction

Tight junctions between endothelial cells, composed primarily of claudin-5, occludin, and junctional adhesion molecules linked to actin via ZO-1, create the primary physical barrier. These junctions minimize the paracellular space, allowing only tiny hydrophilic molecules (<1.8 nm diameter) to enter, with negligible entry [4,26]. Claudin-5 deletion demonstrates selective permeability to molecules < 800 Da, reinforcing the tight junctions’ role in size-dependent exclusion [27,28,29]. Consequently, more than 98% of small-molecule drugs fail to cross the intact blood–brain barrier (BBB) passively [1,2,30]. The integrity of tight junctions can be disrupted by inflammation or hypoxia, transiently increasing permeability, whereas the tightening of tight junctions correlates with BBB maturation [31]. Conversely, in development, the tightening of tight junctions (especially claudin-5 enrichment) correlates with the maturation of BBB function [29,32]. Overall, the tight junctions network endows the BBB with a restrictive paracellular gate that, in healthy states, admits virtually no polar molecules larger than a few hundred daltons.

The BBB also features biochemical defenses: efflux transporters and metabolic enzymes. P-glycoprotein (P-gp) and breast cancer resistance protein (BCRP), abundant on endothelial membranes (P-gp: ~4–7 pmol/g, BCRP: ~8–16 pmol/g), actively extrude xenobiotics, significantly restricting CNS entry (Storelli et al., 2021). Impaired efflux activity in Alzheimer’s disease reduces clearance of neurotoxic peptides despite normal transporter levels [33]. Brain endothelial cells also express enzymes (γ-glutamyl transpeptidase, alkaline phosphatase, MAO, and cytochrome P450s) that degrade circulating molecules, thereby preventing CNS entry. Thus, tight junctions, efflux transporters, and enzymatic activity collectively ensure that the BBB effectively excludes nearly all drugs and biomolecules from passive entry into the CNS [34,35].

### 2.3. Major Transport Mechanisms Across the BBB

Despite its stringent selectivity, the BBB allows regulated molecular transport via several distinct mechanisms. Passive diffusion enables the transport of a limited range of small (<400–500 Da), lipophilic molecules, including gases (O_2_, CO_2_) and certain drugs (such as nicotine and ethanol). However, only approximately 2–6% of small-molecule therapeutics penetrate the BBB passively due to the stringent physicochemical constraints [2,34]. Tight junctions (~1–2 nm pore size) severely restrict paracellular transport between endothelial cells, permitting only negligible passage of water and small ions. Pathological disruption of tight junction integrity can temporarily increase permeability to molecules that are otherwise restricted [4,27,28]. Carrier-mediated transport (CMT) involves specialized solute transporters that facilitate the uptake of essential nutrients, such as glucose (GLUT1, ~0.5 μmol/g/min), amino acids (LAT1), and vitamins [36]. Notably, alterations in transporter expression occur in diseases such as Alzheimer’s [33]. Receptor-mediated transcytosis (RMT) utilizes specific endothelial receptors (e.g., TfR, LDLR, LRP-1, insulin receptor) to shuttle essential peptides, proteins, and nutrients across the BBB. Although effective, RMT typically delivers limited amounts (~0.1–2% of the injected dose for therapeutic antibodies), sufficient for potent drugs [37,38]. Adsorptive-mediated transcytosis (AMT) involves non-specific electrostatic interactions with cationic or amphipathic molecules, significantly enhancing uptake (e.g., ~10–20-fold increase for cationic albumin). Despite its non-selectivity, AMT is exploited therapeutically, though its potential cytotoxicity warrants careful control [39]. Table 1 summarizes the key features of each central transport mechanism across the BBB, highlighting representative molecules, size limitations, typical efficiencies, and relevant examples.

Given the complexity and strictness of BBB physiology, selecting appropriate experimental models is critical for evaluating nanocarrier transport, biodistribution, and therapeutic efficacy. Table 2 provides a comparative overview of in vitro and in vivo models for studying nanotechnology transport across the BBB. Each model offers physiological relevance, experimental control, and a balance of throughput. In vitro models range from simple immortalized cell monolayers with high scalability but low barrier tightness to advanced hiPSC-derived BBB-on-chip systems that closely recapitulate human BBB properties (with TEER values approaching in vivo levels) [40,41]. Incorporating astrocytes, pericytes, or flow typically enhances barrier integrity and transporter functionality, albeit with increased complexity. In vivo rodent models remain indispensable for demonstrating NP delivery in an intact organism, as they provide the full context of the neurovascular unit and pharmacokinetic realism. However, species differences (e.g., rodent vs. human P-gp expression) must be taken into account [42]. Non-human primates offer the most human-like BBB for late-stage validation, although practical and ethical constraints limit their use. Notably, emerging zebrafish models can expedite early screening, but results should be extrapolated to mammals with caution. In summary, no single model is “best” for all purposes—researchers should choose the model that best fits their experimental goals. Early high-throughput screens can be performed in simpler systems (cell lines or zebrafish), medium-complexity models (primary co-cultures, iPSC chips) are ideal for mechanistic insights, and critical translational studies are reserved for rodent and primate models. This strategic, multi-model approach maximizes discovery efficiency and clinical relevance, guiding nanomedicine development across the BBB toward successful human applications.

## 3. Key NP Properties for Crossing the BBB

**Figure 2:**
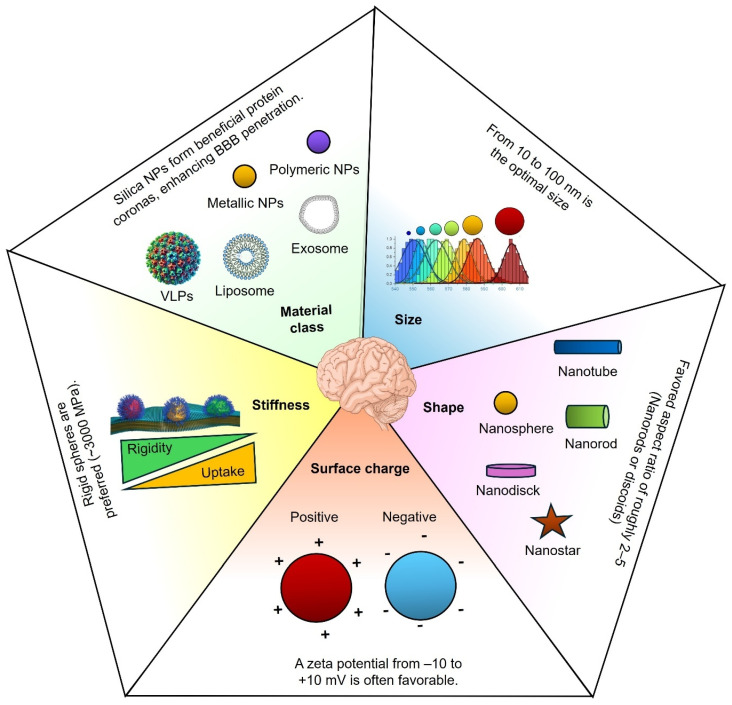
Key physicochemical parameters influencing nanoparticle transport across the BBB. Effective BBB penetration by nanocarriers depends on the optimization of multiple design parameters. Material class influences biodistribution and immune interactions; VLPs, liposomes, exosomes, and polymeric or metallic nanoparticles exhibit distinct advantages. A size of 10–100 nm is typically optimal for transcytosis. Shape affects cellular uptake and biodistribution, with nanorods and nanotubes (aspect ratio ~2–5) showing enhanced BBB permeability. Surface charge modulates electrostatic interactions with the endothelium; a near-neutral zeta potential (−10 to +10 mV) minimizes opsonization and promotes translocation. Stiffness also plays a critical role; rigid particles (Young’s modulus > 3000 MPa) tend to favor endothelial uptake over softer counterparts. The rational tuning of these parameters is essential for the design of CNS-targeting nanomedicines. Adapted from [58,59].

Delivering therapeutics across the BBB requires NPs with finely tuned physicochemical properties. Size, surface charge, shape, and composition determine a nanocarrier’s ability to traverse the brain endothelium while evading clearance [15,57]. Recent preclinical studies (2020–2025) provide quantitative insights that guide the rational design of BBB-penetrant nanotechnologies, as summarized in Figure 2.

### 3.1. Physicochemical Properties

*Size:* NP size profoundly influences BBB transport. Optimal diameters typically range from tens to hundreds of nanometers, striking a balance between efficient transcytosis and minimal sequestration by clearance organs. In a mouse model, 20 nm insulin-coated gold NPs (AuNPs) achieved the highest brain accumulation (at 2 h post-injection) compared to identical 50 nm and 70 nm formulations. The 20 nm AuNPs showed the widest biodistribution in brain tissue. In contrast, larger AuNPs were less effective [60]. Similarly, an in vitro BBB model demonstrated that 25 nm PEGylated silica nanoparticles exhibited higher transport efficiency compared to 50 nm and 100 nm nanoparticles under the specific experimental conditions evaluated [61]. In a rodent brain tumor model, doxorubicin-loaded PEGylated silica nanoparticles (~25 nm in size, with a slight positive surface charge) remained in circulation for over 24 h. They achieved ~6-fold higher accumulation of the drug in the brain compared to free doxorubicin [62]. However, it is important to note that such pronounced results were observed under the specific experimental conditions of that study (using that particular nanoparticle formulation) and cannot be directly generalized across all drug delivery contexts [62]. These small NPs likely exploit transcellular pathways and narrow paracellular gaps between endothelial cells, whereas tight junctions mostly exclude larger particles [63,64].

There are practical size limits at both extremes. Particles < 10 nm are rapidly filtered by the kidneys and can diffuse out of the brain almost as quickly as they enter. For instance, ultrasmall (~2 nm) gold nanoclusters readily cross the BBB but exhibit non-negligible neurotoxicity and rapid clearance [65,66]. On the other hand, NPs larger than 200 nm are prone to opsonization and sequestration by the reticuloendothelial system (RES) in the liver and spleen, resulting in an insufficient fraction reaching the brain. Indeed, 200–250 nm gold NPs were found to accumulate less in the brain (and in systemic organs) than 10–15 nm gold NPs, which distributed more broadly (albeit with higher potential toxicity) [66,67]. These studies indicate an optimal NP size window (roughly 10–100 nm) that maximizes BBB penetration. Within this window, particles of 50–150 nm often achieve longer plasma half-lives than those of smaller sizes yet are still small enough to extravasate; for example, ~50 nm polymeric NPs showed deeper brain tissue penetration in a brain injury model than 200 nm or 800 nm analogues.

Additionally, particle size has a strong influence on the protein corona composition in circulation, which in turn impacts BBB receptor-mediated uptake. For instance, lipid NPs (~30 nm) preferentially bind apolipoprotein E, enhancing LDLR- and LRP-1-mediated transcytosis compared to larger counterparts. Notably, the optimal nanoparticle size range may vary under pathological conditions; for instance, polymeric nanoparticles of approximately 100 nm exhibited greater accumulation in GBM models compared to smaller or larger particles, suggesting size-dependent performance specific to this context [68,69]. Thus, the ideal size range of ~10–100 nm balances efficient receptor targeting, prolonged systemic circulation, and enhanced brain delivery.

*Surface charge (zeta potential):* The BBB endothelium carries a negatively charged glycocalyx, meaning NP surface charge critically mediates electrostatic interactions with the barrier. Mildly cationic NPs can undergo enhanced adsorptive uptake by BBB cells; however, a strongly positive charge also risks disrupting the integrity of tight junctions. Quantitative perfusion studies have shown that neutral or slightly anionic surface charges are optimal for safe BBB transit. Lockman et al. found that neutral NPs and low-concentration anionic formulations preserved rat BBB integrity and achieved brain delivery, whereas highly cationic NPs caused immediate BBB leakage and toxicity. Notably, the brain uptake rate of anionic NPs (at non-disruptive concentrations) exceeded that of neutral or cationic versions in the same model [70]. This suggests a trade-off: positively charged NPs bind avidly to the negatively charged cell membranes, promoting internalization, but excessive positive charge triggers unwanted barrier opening and clearance by proteoglycan-rich regions of the endothelium.

Recent mechanistic studies support a moderate cationic charge for BBB crossing. A 2020 mathematical model incorporating endothelial surface charge predicted that a positively charged NP experiences enhanced transcellular permeability by electrostatic attraction to the negatively charged membrane [71]. Experimentally, cationic liposomes (+30 mV ζ-potential) show higher uptake by brain endothelial cells than equivalent neutral or −30 mV liposomes [9,72]. In one comparative study across eight cell lines, positively charged NPs exhibited the fastest uptake rates compared to negative or neutral NPs [73]. However, high uptake does not guarantee deep brain delivery. Strong cationic particles may stick to the luminal surface or become entrapped in endothelial lysosomes. In contrast, negatively charged NPs (e.g., ~−35 mV) diffuse more freely and can penetrate further into brain parenchyma [9,74]. For example, one report noted that although +30 mV gold NPs enter BBB cells efficiently, −36 mV gold NPs penetrated brain tissue due to greater mobility through the endothelium [72]. The downside is that negative NPs (and uncharged PEGylated NPs) tend to be less readily internalized. In vivo, neutral or PEG-coated NPs often exhibit the longest circulation times; for instance, neutral PEGylated liposomes can circulate for hours. In contrast, highly cationic NPs are rapidly opsonized and cleared from the bloodstream [75]. Slightly negative or near-neutral surfaces appear optimal for balancing efficient BBB uptake and systemic circulation. Typically, a zeta potential within ±10 mV is favorable for BBB-targeted nanocarriers, as it offers adequate interaction with the negatively charged endothelial surface while minimizing cytotoxicity and rapid clearance. Notably, surface charges exceeding ±25 mV can compromise vascular integrity and trigger undesirable BBB leakage. Thus, carefully tuning nanoparticle surface charges, potentially employing zwitterionic or charge-shielding coatings that selectively become positively charged only near the endothelial membrane, is a strategic approach to maximize effective brain delivery while preserving BBB integrity [70]. 

*Shape:* Whether spherical, rod-like, discoidal, or filamentous, the shape of NPs has emerged as a critical design parameter for BBB transit. Shape affects how particles navigate blood flow, how they interface with cell membranes, and how phagocytes recognize them. Recent studies have shown that non-spherical shapes can enhance brain delivery under specific conditions. For instance, polymeric nanorods targeted to transferrin receptors on BBB endothelium exhibited a seven-fold higher cellular uptake than equivalent spheres in an in vitro BBB model [76]. Although that dramatic seven-fold uptake was reported in an earlier study with functionalized rods, even untargeted rods demonstrate advantages in transcytosis efficiency. Nowak et al. (2020) used a human microfluidic BBB model to compare 200 nm spheres with rod-shaped particles. While spherical NPs adhered more to the endothelial surface, the rod-shaped NPs translocated across the endothelium approximately twice as efficiently per cell-bound particle. The authors hypothesized that rods enter endothelial cells via a distinct pathway or orientation that favors vesicular transport [67,77]. In essence, a rod or filamentous shape can “partition” into the cell membrane differently than a sphere, potentially exploiting elongated endocytic pits or aligning with membrane invaginations.

Particle flexibility often goes hand-in-hand with shape. Filamentous micelles and worm-like filomicelles (long and flexible) have demonstrated extraordinarily long circulation and increased accumulation in some tissues, likely by evading phagocytosis. In the brain context, one benefit of discoidal or rod-shaped NPs is improved margination in capillaries; these shapes drift toward the endothelial wall under flow more than spheres, increasing the odds of BBB contact. A 2024 study by Sierri et al. directly compared ~100 nm lipid NPs of identical chemistry but different shapes (spherical vs. discoid vs. deformable “soft” particles) in a human BBB model. Strikingly, discoidal NPs had about double the endothelial permeability of spheres (permeability coefficient ~1.3 × 10^−5^ cm/min for discoids vs. ~6–7 × 10^−6^ for spheres). The deformable (ellipsoidal) NPs were intermediate in BBB crossing. The discoids also traversed intercellular tunneling nanotubes more efficiently, suggesting that shape influences initial BBB crossing and subsequent spread in brain tissue. The enhanced BBB transit observed for discoidal nanoparticles was proposed to result from their larger surface area, which is available for interactions with the cell membrane, and their potentially favorable orientation during cellular trafficking [78]. Consistent with this, Fu et al. (2022) found that rod-shaped polymeric NPs outperformed spherical NPs in crossing an in vitro brain microvascular model [79]. In summary, elongated or flattened shapes (rods, disks) promote BBB interaction and transcytosis, whereas spherical NPs often remain in circulation or are taken up by the RES. That said, extremes of shape can affect biodistribution: high-aspect-ratio filaments might avoid uptake by liver/spleen but could be too large to traverse tight endothelial junctions if very long. Thus, an aspect ratio of roughly 2–5 (standard for short rods or discoids) appears optimal for BBB delivery [67]. By tailoring NP shape, researchers can influence circulation half-life (with filaments lasting longer in blood) and enhance margination to brain capillaries, thereby improving the chances of BBB penetration.

*Stiffness:* NP stiffness has a significant influence on BBB permeability and transcellular transport efficiency. Generally, stiffer NPs exhibit greater endothelial uptake and enhanced BBB penetration compared to softer particles, as evidenced by rigid polystyrene spheres (bulk modulus ~3000 MPa) demonstrating approximately tenfold higher translocation than softer PEG-based hydrogels (~3 MPa) [67,80,81,82].

In vivo GBM models confirm that there is a greater accumulation of stiffer NPs within brain tumors compared to their deformable counterparts [72]. Mechanistically, stiffer particles are more readily endocytosed due to lower membrane deformation requirements, although softer NPs may undergo more efficient intracellular trafficking and exocytosis, facilitating transcytosis. Stiffness also influences vascular margination, where rigid particles migrate toward vessel walls, thereby increasing endothelial interactions without disrupting the integrity of tight junctions [67]. Thus, NP stiffness modulation represents a promising strategy for optimizing drug delivery across the BBB.

### 3.2. Composition and Material Class

The composition of NPs (lipidic, polymeric, protein-based, inorganic) significantly influences their BBB permeability and biocompatibility.

*Lipid-based NPs:* Liposomes and solid lipid NPs (SLNPs) effectively cross the BBB, particularly when functionalized. PEGylated liposomes (~100 nm) have limited penetration on their own, but ligand decoration significantly enhances uptake. For instance, VCAM-1 peptide-targeted liposomes achieved a ~6% injected dose/g brain versus a ~1.2% untargeted dose [83]. Thiamine-functionalized SLNs also increased brain drug concentrations 1.4-fold [84]. Lipid NPs excel in genetic cargo delivery, showing efficient mRNA transfection with reduced off-target accumulation [75].

*Polymeric NPs:* Biodegradable polymers (PLGA, PLA, dendrimers) commonly enhance BBB transport through PEGylation or the use of targeting ligands. LDL receptor-targeted PLGA NPs achieved a ~4% ID/g brain concentration compared to a ~1% ID/g concentration for untargeted NPs [24,85]. Polymeric nanocarriers offer tunable release kinetics and flexible shapes, enhancing penetration and tumor suppression [67].

*Protein-based NPs:* Ferritin and virus-like particles (VLPs) naturally exploit receptor-mediated pathways. Human H-ferritin effectively delivers antibodies into brain tumors [86]. Peptide-functionalized protein NPs (e.g., RVG29, transferrin, angiopep-2) further enhance receptor-specific uptake [87].

*Inorganic NPs:* Gold, iron oxide, and silica NPs provide stable cores suitable for functionalization and imaging. Targeted gold NPs (~50 nm) significantly outperform untargeted ones (0.23% vs. 0.04% ID/g) [66]. Iron oxide nanoparticles (SPIONs, <20 nm) achieve brain accumulation suitable for MRI imaging when suitably coated [24]. Moreover, an “optimal” iron oxide size of around 10–50 nm has been identified to maximize circulation time and minimize phagocytic uptake [88]. Silica NPs (~25 nm) form beneficial protein coronas, enhancing BBB penetration [62].

*Poly(propylenimine) (PPI/DAB) dendrimers:* Evidence from in vivo studies indicates that PPI can traverse the blood–brain barrier (BBB) via adsorptive-mediated transcytosis (AMT) of cationic surfaces and via receptor-mediated transcytosis (RMT) when decorated with transferrin receptor (TfR) or LRP1 ligands. Design-relevant parameters include dendrimer generation (G3–G6), terminal groups (native –NH_2_ vs. sugar/PEG/histidine–maltose shells), and the hydrodynamic size and ζ-potential of drug/nucleic-acid (NA) dendriplexes (typically ~120–180 nm; surface charge often shielded toward near-neutral in serum). Representative in vivo data show that transferrin–PPI G3 dendriplexes deliver plasmid DNA to mouse brain with >2-fold higher gene expression than untargeted controls after intravenous administration [89]; histidine–maltose–coated PPI G4 increases brain levels after intranasal dosing and preserves cognition in an Alzheimer’s disease (AD) mouse model [90]; and Angiopep-2–PEG–PPI formulations enhance LRP1-mediated glioma targeting and chemotherapeutic delivery [91].

Table 3 summarizes the main advantages and limitations of NP materials commonly explored for brain drug delivery across the BBB. Lipid-based NPs, including liposomes and solid lipid nanoparticles, offer excellent biocompatibility, effective ligand-mediated BBB crossing, and efficient genetic cargo delivery but face challenges related to stability and rapid clearance by the reticuloendothelial system (RES). Polymeric NPs (e.g., PLGA, PLA) are FDA-approved and highly biocompatible, featuring tunable release profiles and easy surface functionalization; however, they can accumulate due to incomplete biodegradation, potentially inducing cytotoxicity, and require optimization for effective passage across the BBB. Dendrimers, such as poly(propylenimine) (PPI) and PAMAM, utilize multivalent transcytosis pathways, exhibit excellent targeting potential, and demonstrate therapeutic efficacy in central nervous system (CNS) models; nonetheless, their clinical translation is limited by intrinsic cytotoxicity, immunogenicity, and poor biodegradability. Protein-based NPs (ferritin, albumin, and virus-like particles) naturally exploit receptor-mediated transport mechanisms, provide superior biocompatibility, and facilitate versatile ligand conjugation; however, they have constrained payload capacity, stability issues during formulation or storage, and immunogenic risks upon repeated administration. Inorganic NPs (gold, iron oxide, and silica) are structurally stable, multifunctional (therapeutic/imaging capabilities), and easily functionalized for targeted delivery; nevertheless, their non-degradable nature leads to organ accumulation, potential chronic toxicity, and limited brain penetration unless optimized through size reduction (<10 nm) or targeted strategies like magnetic guidance or ultrasound-mediated BBB disruption.

Table 4 presents an overview of recent experimental findings from original research on NP performance in brain delivery. It includes key physicochemical attributes (size, charge, and composition), quantitative metrics on brain accumulation (% ID/g), circulation half-life, and therapeutic outcomes across both healthy and pathological BBB models. By contrasting NP types, lipid-based, polymeric, protein-based, and inorganic, under varied surface modifications and targeting strategies, this table is a critical reference for guiding the rational design of nanocarriers optimized for BBB penetration. Overall conclusions highlight that NP functionalization using BBB receptor-specific antibodies or appropriate polymer coatings significantly enhances brain uptake compared to untargeted particles. Optimal performance is typically achieved with NP sizes below 100 nm, which carry neutral or slightly negative surface charges, facilitating longer circulation half-lives and improved brain retention. However, functionalization alone does not guarantee therapeutic efficacy universally; thus, surface modifications and particle composition must be carefully tailored to maximize clinical outcomes.

## 4. Nanotechnological Strategies for Crossing the BBB

We focused on primary studies published between 2020 and 2025 that reported quantitative brain uptake metrics (e.g., %ID/g), model fidelity indicators (e.g., TEER values, transporter profiling), and translational endpoints (e.g., survival, functional recovery), while using recent reviews (2020–2024) as a complementary foundation. Inclusion criteria were the explicit reporting of nanoparticle physicochemical properties (size, PDI, ζ-potential), administration route and dose, sampling time points, and quantification methods. Studies that did not meet these standards were excluded or considered only qualitatively.

**Figure 3:**
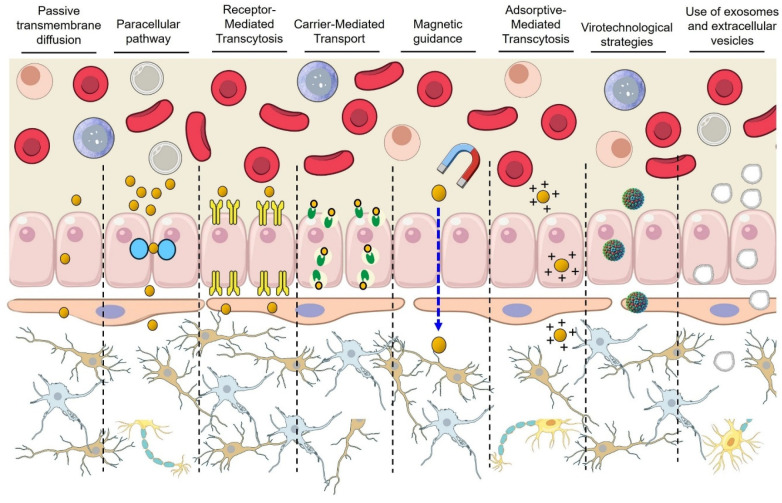
Mechanisms of nanoparticle transport across the BBB. NPs can traverse the BBB via multiple pathways, each with distinct biophysical and biochemical requirements. These include: (1) passive transmembrane diffusion, mainly for lipophilic, low-molecular-weight compounds; (2) p transport, which is highly restricted by tight junctions; (3) receptor-mediated transcytosis, utilizing ligands that bind to endothelial receptors (e.g., transferrin, insulin); (4) carrier-mediated transport, which exploits endogenous solute transporters; (5) magnetic guidance, where externally applied magnetic fields steer magnetic nanoparticles; (6) adsorptive-mediated transcytosis, driven by electrostatic interactions between cationic nanocarriers and negatively charged endothelial membranes; (7) virotechnological strategies, leveraging viral vectors or virus-like particles for efficient delivery; and (8) the use of exosomes and extracellular vesicles, which offer endogenous transport capabilities and biocompatibility. Modified from [12,115,116].

The BBB severely restricts brain uptake of systemically administered therapeutics. Innovative nanotechnologies are being developed to ferry drugs across the BBB in meaningful quantities (Figure 3). Recent studies emphasize human-relevant models to assess translational potential, from induced pluripotent stem cell (iPSC)-derived BBB cultures to non-human primates and early clinical trials. Below, we review key nanotechnological strategies for BBB crossing, highlighting quantitative brain delivery metrics (e.g., percentage of injected dose per gram brain, brain–plasma ratios) and therapeutic outcomes (tumor suppression, cognitive improvements) where available.

### 4.1. Receptor-Mediated Transcytosis (RMT)

RMT exploits endogenous nutrient/carrier receptors on brain endothelium to shuttle NPs or biologics into the brain. Binding to receptors such as the transferrin receptor (TfR), insulin receptor (INSR), low-density lipoprotein receptor-related protein 1 (LRP1), lactoferrin receptor, or folate receptor triggers internalization and vesicular transport across endothelial cells. This strategy has dramatically improved BBB delivery in multiple models:

*Transferrin Receptor (TfR):* The TfR is abundantly expressed on brain microvessels (especially during development) and is a prime RMT target. Classical studies in rats showed that an anti-TfR antibody (OX26) achieved ~0.3% of the injected dose (ID) in the brain, over 10-fold higher than a non-specific IgG (0.03% ID) [117]. Recent work has refined TfR-targeting by adjusting antibody affinity/valency. Johnsen et al. demonstrated that gold NPs conjugated with a low-affinity, monovalent anti-TfR delivered 0.23% ID/g into brain parenchyma, versus only 0.04–0.08% ID/g with high-affinity bivalent variants [24]. Likewise, Denali Therapeutics engineered a TfR-binding “Enzyme Transport Vehicle” (ETV) with moderate affinity; in mice, an ETV–IDS (iduronate-2-sulfatase) fusion achieved broad CNS distribution and enzyme uptake by neurons, lowering pathological glycosaminoglycans in the brain by 49–76%, compared to only 25–43% reduction using a high-affinity bivalent IgG format. Notably, monovalent TfR carriers avoid “trapping” in endothelial endosomes, yielding higher transcytosis efficiency [118]. In non-human primates, systemically delivered TfR-targeted biologics have demonstrated distributed brain delivery without significant peripheral side effects, validating TfR RMT as a clinically viable route. For example, a bispecific antibody with one TfR-binding arm and one therapeutic arm reached ~0.5–1.1% ID/g in capillaries and significantly enhanced parenchymal delivery [24]. These advances underscore that binding affinity and avidity are critical: too high an affinity can impede release into brain parenchyma, whereas optimized moderate affinity promotes deeper delivery.

*Insulin receptor (INSR):* The INSR is widely expressed on the BBB endothelium and can mediate brain uptake of insulin and analogues [100]. High-affinity human INSR antibodies (e.g., 83-14) have been used as “Trojan horses” to ferry drugs across the BBB. In a clinical trial for Hunter syndrome, an INSR-targeted enzyme (valanafusp alpha) showed CNS activity, indicating successful BBB penetration [104]. NPs have also leveraged INSR; for instance, HSA (human serum albumin) NPs conjugated with an insulin mimetic (29B4 antibody) penetrated the BBB and elicited CNS therapeutic effects in rodents. Although INSR targeting can risk hypoglycemic signaling, careful engineering (e.g., insulin agonist antibodies that activate transport without strong insulin-like effects) has enabled significant brain delivery [119,120].

*LRP1 (Low-Density Lipoprotein Receptor-Related Protein 1):* LRP1 is highly expressed on the brain endothelium and shuttles ligands such as ApoE and lactoferrin. The peptide Angiopep-2 (Ang-2) binds LRP1 and has been widely used to decorate NPs. Angiopep-functionalization triggers robust transcytosis; an Ang-2–drug conjugate crossed the BBB and accumulated in brain tumors in clinical trials (ANG1005 for glioma). Recent docking studies have optimized Ang-2 analogues for tighter LRP1 binding [121,122,123]; an Ang-2–drug conjugate crossed the BBB and accumulated in brain tumors in clinical trials (ANG1005 for glioma). Recent docking studies have optimized Ang-2 analogues for tighter binding to LRP1 [124]. In one study, an artificial LRP1-binding peptide, “L57,” demonstrated efficient uptake in primary human brain microvascular endothelial cells, suggesting it may serve as a novel BBB shuttle [125]. Tubular transcytosis pathways have been observed for LRP1, suggesting unique mechanisms that can be modulated by peptide “shuttles” [126].

*Lactoferrin receptor:* Lactoferrin (Lf), an iron-binding glycoprotein, crosses the BBB via its receptor on brain endothelium [127,128]. NPs coated with lactoferrin exploit this route. For example, Youssef et al. (2025) coated lipid nanocapsules with Lf to deliver the antioxidant drug apocynin (APO) and lavender oil in a rat epilepsy model. The Lf-coated nanocapsules achieved an increase in brain accumulation of APO and significantly reduced seizure severity, yielding a Racine score of ~0.67 (near absence of seizures) versus much higher scores in uncoated-NP or free-drug groups. Lf-NPs also prolonged seizure latency and lowered neuroinflammatory markers, indicating effective therapeutic delivery to the brain [128]. Similarly, oral Lf-decorated gold NPs have been studied for GBM, given that LfR is upregulated in many tumors [129]. These results highlight lactoferrin’s potential as an endogenous targeting ligand to improve drug BBB permeability.

*Folate receptor:* The folate receptor-α is minimally expressed in the endothelial cells forming the intact BBB and is predominantly localized within the choroid plexus. However, it is often significantly upregulated in brain tumor cells, such as gliomas, and metastatic or leptomeningeal tumor deposits [130]. Due to this selective overexpression, folic acid has become a widely employed targeting ligand for nanoparticles [100], facilitating receptor-mediated endocytosis specifically into tumor tissues. For example, folate-coated gold nanoparticles loaded with indomethacin demonstrated enhanced uptake in folate receptor-positive glioma cells, resulting in prolonged survival in glioma-bearing mouse models [131,132]. Thus, although folate receptor-mediated transcytosis is not a significant pathway across a healthy, intact BBB, it can effectively mediate selective transport into brain tumor tissues, providing a tumor-specific BBB bypass without substantial impact on healthy brain tissue.

To maximize RMT delivery, researchers combine ligands (e.g., a TfR antibody with a cell-penetrating peptide or an RVG peptide) on the same NP [87]. These multi-target NPs aim to engage multiple uptake pathways synergistically. Overall, RMT-based nanocarriers have achieved measurable brain uptake on the order of 0.1–0.5% ID/g in rodents [133], which, while modest in absolute terms, represents an order-of-magnitude improvement over untargeted delivery. More importantly, these carriers have demonstrated therapeutic efficacy in disease models, ranging from enzyme replacement in lysosomal diseases (reducing CNS pathology) to drug delivery in brain tumors (inhibiting growth), underscoring the translational promise of RMT nanotechnologies [118].

### 4.2. Adsorptive-Mediated Transcytosis (AMT)

Adsorptive-mediated transcytosis relies on the electrostatic attraction of cationic molecules to the negatively charged endothelial membrane, inducing nonspecific endocytosis. Unlike RMT, AMT does not require specific receptors, making it a broadly applicable technique [134]. Various cationic coatings and cell-penetrating peptides (CPPs) have been used to trigger AMT for brain delivery:

*Cationic cell-penetrating peptides:* Poly-arginine peptides (such as R9), the TAT peptide (derived from the HIV transactivator protein), and penetratin (from Antennapedia) carry multiple positive charges that promote adsorption to the BBB surface. For example, NPs functionalized with PepH3, a 7-amino-acid cationic peptide derived from dengue virus capsid, showed greatly enhanced uptake in BBB models. PepH3-tagged NPs exhibited active transcytosis across both rat and human BBB cell monolayers [135,136]. In vivo, radiolabeled PepH3 derivatives achieved high brain uptake with low accumulation in liver, lung, and kidney, indicating a degree of selectivity [135,137]. Notably, making the endothelial surface less negative (by enzymatically removing glycocalyx sialic acids) reduced PepH3-NP uptake, confirming that electrostatic interactions are the primary driver. These findings show that cationic shuttles can exploit AMT to cross the BBB, and some (like PepH3) may do so with minimal off-target deposition [136]. Other CPPs, such as penetratin and poly(arginine)-8, have similarly increased NP translocation in vitro and in situ (e.g., brain perfusion models) by several-fold compared to unmodified NPs. However, in vivo quantitation is less common.

*Cationic polymers (e.g., chitosan):* Chitosan, a polycationic polysaccharide, has been widely used to decorate NPs for BBB delivery, as recently reviewed by [138]. Its positive charge and mucoadhesive properties facilitate both the absorption of AMT and the modulation of tight junctions. Khan et al. (2023) demonstrated that DNA-loaded chitosan nanoparticles (NPs, ~260 nm, with a positive ζ potential) could effectively transfect brain cells in vivo after systemic administration. In their study, GFP-encoded chitosan NPs injected intraperitoneally in mice showed GFP expression in the brain, confirming that the NPs crossed the BBB. The brain delivery was achieved without the use of chemical targeting ligands; the chitosan’s inherent AMT property was sufficient. Notably, the chitosan vectors showed low cytotoxicity and immunogenicity in vitro (U87 glioma cells had ~85% viability) and no apparent toxicity in vivo [139]. These data position chitosan NPs as safe, efficient gene delivery vehicles to the CNS, leveraging adsorptive uptake. Other cationic polymers (e.g., polyethyleneimine, cationic dendrimers) also promote BBB transit, though toxicity must be carefully managed.

*Cationic surface coatings:* Even without distinct peptides or polymers, tuning an NP’s surface charge can impact BBB uptake. Slightly positive or even “near-neutral” (mildly negative) zeta potentials favor BBB transcytosis [133]. For instance, one study found that rod-shaped polymeric NPs with a mildly negative surface had optimal uptake in brain endothelial cells (7-fold higher than neutral NPs) [76,140]. In practice, some researchers coat NPs with cell membranes (from, e.g., leukocytes or platelets) to confer biological identity and charge that enhance BBB passage via a combination of AMT and other mechanisms. The general principle is that increasing NP affinity for the endothelial membrane (through charge or hydrophobic patches) can initiate vesicular transport across the BBB.

Overall, AMT-based approaches often lack the absolute specificity of RMT, but they offer versatility. They are beneficial for delivering large macromolecular complexes or gene vectors that might not fit into a single receptor pathway. By combining AMT peptides with targeting ligands (dual-function NPs), researchers aim to achieve both high uptake and specific delivery. The primary quantitative limitation of AMT is potential sequestration in endothelial cells or perivascular spaces; however, evidence from studies, such as the chitosan NP study, suggests that a meaningful fraction of the dose can reach the parenchyma to exert biological effects.

### 4.3. Magnetically Guided NPs

Magnetic targeting employs external magnetic fields to direct superparamagnetic iron oxide NPs (SPIONs) across the BBB, concentrating them in specific brain regions such as tumors. SPIONs coated with surfactants (e.g., Tween) have successfully crossed the intact BBB in rats when guided by magnets, with negligible uptake observed without magnetic fields [141,142,143].

*Magnetic liposomes:* In glioma therapy, magnetic liposomes containing temozolomide and SPIONs demonstrated significant tumor accumulation, slowed tumor growth, and modestly improved survival under magnetic guidance compared to controls. These NPs also acted as MRI contrast agents without observed toxicity [142].

*Magnetic hyperthermia combo:* Combining magnetic targeting with hyperthermia via alternating magnetic fields (AMF) further enhances therapeutic outcomes. A recent study utilized Fe_3_O_4_@Chitosan@ZIF-8@RVG29 nanoparticles, resulting in the effective killing of glioma cells and tumor apoptosis [144].

Magnetic targeting significantly increases drug concentration and spatial precision in brain tumors. Despite requiring specialized equipment and facing limitations in magnetic field penetration, ongoing clinical trials suggest this approach as a promising strategy for overcoming the BBB.

### 4.4. Virotechnological Strategies

Viruses naturally evolved to penetrate biological barriers, including the BBB (some neurotropic viruses cross via receptor-mediated mechanisms). Engineered viral vectors and virus-like particles leverage this capability for drug and gene delivery.

*Adeno-Associated Viruses (AAV):* AAV vectors (∼25 nm) are leading vehicles for gene delivery. Specific serotypes (e.g., AAV9) can cross the BBB, especially in neonatal mice or when the BBB is mildly perturbed. However, BBB transduction by natural AAVs is very limited in adult primates. Breakthrough work from 2020 to 2023 has resulted in engineered AAV capsids with enhanced blood–brain barrier (BBB) penetration. Chuapoco and colleagues developed AAV.CAP-Mac, which was selected by iterative screening for marmosets and macaques. In adult rhesus monkeys, AAV.CAP-Mac achieved widespread brain transgene expression after IV injection, transducing ~1.3% of cortical neurons, compared to only 0.5% with conventional methods. AAV9.CAP-Mac showed broad tropism (neurons and some glia) and delivered functional genes (e.g., GCaMP for neural imaging) across multiple brain areas [145]. Another group created AAV-F (AAV.PHP.eB variants)* that crosses the BBB in mice by interacting with LY6A on endothelial cells, though these do not translate to primates. In 2023, an AAV capsid was reported that binds human TfR1 to ferry genes across the BBB. Such TfR-targeted AAVs increased mouse CNS gene delivery and showed uptake in ex vivo human BBB models [146]. The quantitative gains are striking; one variant (AAV.CAP-B10) showed 3–4 times higher neuronal transduction in macaques than AAV9, with much lower liver off-targeting. These advances suggest that IV AAV gene therapy for the brain is becoming a feasible option. Indeed, systemic AAV9 is already used in infants (e.g., onasemnogene for SMA) to deliver genes to spinal motor neurons, leveraging a partially immature BBB. Ongoing clinical studies are exploring AAV capsids for adults with Alzheimer’s and Parkinson’s, aiming to achieve a few percentage points of CNS targeting, which is enough to achieve a therapeutic benefit in many cases [145].

*Lentiviral and other viral vectors:* Lentiviruses (e.g., modified HIV-1) and adenoviruses can transduce brain cells but generally do not cross the BBB efficiently with IV. Hence, they have been used via direct injection into the brain or CSF. Recent innovations involve coating or retargeting these larger viruses. For example, a lentivirus pseudotyped with rabies glycoprotein (RVG) was shown to reach CNS neurons from the bloodstream in mice, using RVG’s ability to interact with nicotinic acetylcholine receptors on the BBB endothelium [144]. Another approach uses exosomes to “piggyback” lentiviral vectors (see the hybrid exosome section). While systemic lentiviral delivery to the brain is not yet routine, the field is moving toward producing virus-like particles decorated with BBB-targeting ligands.

*Virus-Like Particles (VLPs):* These are self-assembled protein cages derived from viruses, but without any viral genome, essentially forming “nanocontainers” that can carry drugs or genes. VLPs from various sources have been tested for brain delivery, including bacteriophage Qβ and MS2, plant viruses like Tobacco Mosaic Virus (TMV) and Cowpea Chlorotic Mottle Virus (CCMV), and human virus capsids (e.g., hepatitis B core). VLPs are typically 20–150 nm, a size amenable to crossing fenestrated barriers and, with modifications, potentially the BBB. For instance, TMV nanorods (300 × 18 nm) were albumin-coated to prolong circulation and successfully used to image and treat brain tumors in mice [77,147]. The filamentous shape of TMV may aid its transport along endothelial cells. Qβ VLPs, which are ~30 nm icosahedra, have been functionalized with peptides such as angiopep or transferrin to engage RMT, effectively combining VLPs with the RMT approach. One study reports that exosomes loaded with a VLP carrying rhodamine dye crossed the BBB and increased brain drug delivery [148], illustrating a hybrid of VLP and exosome strategies. While still preclinical, VLPs offer high customizability (both genetic and chemical) to display targeting ligands and can be produced in high yields from plants or bacteria. They also tend to be biodegradable and less immunogenic than whole viruses. The challenge is achieving efficient BBB traversal; current VLPs require surface functionalization. Nonetheless, VLPs are a versatile platform, such as a ~120 nm hepatitis B VLP, which has been used to carry siRNA and target brain metastases, showing improved survival in mice (by homing to a tumor antigen and releasing siRNA upon endocytosis).

In summary, viral vectors have the advantage of active transport mechanisms (receptor- or cell fusion-mediated) that can be highly potent; for example, a single dose of AAV can transduce millions of neurons if it crosses the blood–brain barrier (BBB). The quantitative goal often cited is transducing a few percent points of the total brain cell population or achieving a brain-to-serum drug ratio approaching 0.1; recent viral innovations are closing in on these targets [145]. Meanwhile, non-pathogenic VLPs offer a safer alternative, such as nanocarriers, that can utilize viral entry mechanisms without replicating them. Both are promising for diseases requiring gene therapy, enzyme replacement, or widespread neuromodulation.

### 4.5. Exosomes and Extracellular Vesicles

Exosomes (30–150 nm extracellular vesicles) naturally cross biological barriers and mediate intercellular communication, making them attractive endogenous delivery vehicles for the brain [149]. They possess cell-derived membrane proteins that can confer innate brain tropism; for instance, exosomes from neurons or macrophages may preferentially home to the brain. Key features of exosomes include biocompatibility, low immunogenicity, the ability to be loaded with drugs or RNA, and the flexibility to engineer their surface. Recent developments include the following.

*Intrinsic brain tropism:* Certain exosomes inherently migrate to the brain. MSC (mesenchymal stem cell)-derived exosomes have been shown to cross the BBB in models of stroke and neuroinflammation. In a mouse stroke model, IV MSC exosomes enhanced functional recovery by improving neurogenesis and synaptic remodeling in the brain [150]. This therapeutic benefit implies significant vesicle delivery to brain tissue. Tissue distribution studies often find exosome uptake in the brain to be 2–5-fold higher than equivalent doses of free drug [148]. Moreover, exosomes can cross the BBB without disrupting it; they likely utilize native endocytosis and exocytosis pathways.

*Surface-engineered exosomes:* To enhance targeting, researchers decorate exosome membranes with peptides or antibodies. A prominent example is the exosome displaying the RVG peptide (a rabies virus glycoprotein fragment that binds to nicotinic receptors). Cui et al. (2019) report that RVG-tagged exosomes, loaded with therapeutic cargo, showed enriched delivery to the cortex and hippocampus after IV injection, significantly improving learning and memory in Alzheimer’s mice. In that study, the RVG exosomes (carrying siRNA to knockdown BACE1) restored cognitive function in a murine Alzheimer’s model, whereas non-targeted exosomes had minimal effect. Another group used vesicular stomatitis virus G protein (VSV-G) on exosomes to target brain endothelium, achieving successful gene delivery across the BBB in mice (pseudotyping exosomes with viral fusogens can create a hybrid between viral vector and natural vesicle) [151]. These approaches highlight that by displaying targeting ligands (such as RVG, RGD, and antibodies), exosomes can be directed to specific brain regions or cell types, much like synthetic nanoparticles, but with a biological cloak.

*Drug delivery and hybrids:* Exosomes have been loaded with chemotherapeutics, proteins, or siRNA for brain delivery. Haney et al. demonstrated that exosomes can transport the anti-cancer drug doxorubicin and even large antibodies across the blood–brain barrier (BBB). For glioblastoma, exosomes carrying the antibody cetuximab (targeting EGFR) plus doxorubicin significantly increased antibody brain accumulation and inhibited tumor growth compared to free cetuximab plus the drug. Exosomes delivered more than double the amount of antibody to brain tissue compared to direct antibody injection in that study, due to protected transport and possibly transferable membrane proteins aiding in BBB transit [152]. Beyond using natural exosomes, “hybrid” NPs are being designed, for example, by coating polymeric NPs with exosome membranes (sometimes referred to as cell-derived nanovesicles) to endow them with exosomal homing abilities [148]. These hybrids have shown increased brain uptake and reduced clearance by the mononuclear phagocyte system. One study mentions glioma-targeted exosomes delivering siRNA to suppress oncogenes, crossing the BBB, and prolonging animal survival [153].

Exosome-based delivery is already moving to clinical testing. A first-in-human trial used autologous exosomes loaded with curcumin to treat patients with brain cancer and showed good safety at high doses (though efficacy is still under evaluation). The primary challenges are scalable production and efficient cargo loading. However, since exosomes can be derived from patient cells, they offer a personalized and highly biocompatible therapeutic approach. They naturally avoid P-glycoprotein efflux and can even regulate BBB permeability (some brain endothelial uptake of exosomes triggers signaling that temporarily loosens tight junctions) [149]. With their ability to cross the BBB and deliver functional payloads, e.g., restoring memory in AD models [151], exosomes are poised to become a powerful tool in the neurotherapeutic arsenal.

Active transport nanomotors represent an emerging approach for BBB traversal, with early evidence suggesting significantly improved drug penetration compared to passive methods. The continued development of these active, targeted, and responsive systems holds substantial promise for advancing BBB drug delivery.

Table 5 summarizes nanotechnological strategies for crossing the BBB, detailing delivery vehicles, targeting mechanisms, experimental models, precise quantitative brain uptake metrics, and therapeutic outcomes. Receptor-mediated transcytosis (RMT) is a clinically validated strategy, demonstrating robust brain delivery efficiencies (~0.1–1% ID/g) and significant therapeutic efficacy. Notably, transferrin receptor (TfR)-targeted NPs, including T7-functionalized PLGA NPs, achieve approximately sixfold increases in brain delivery, with measurable improvements in therapeutic outcomes in stroke and glioma models. Angiopep-2-functionalized lipid–silica NPs similarly doubled drug concentrations in the brain, highlighting the advantage of moderate receptor affinity ligands such as LRP1 for brain-targeted therapies.

## 5. Critical Assessment and Future Perspectives of Nanotechnological Delivery Systems Across the BBB

Currently, no commercially available nanoparticle-based products are explicitly approved for actively crossing the BBB following systemic administration. Existing clinical-stage or marketed nanomedicines rely mainly on invasive methods, such as intratumoral or intrathecal administration, or are not explicitly designed for efficient BBB penetration. While nanotechnological delivery systems, including receptor-mediated transcytosis (RMT), adsorptive-mediated transcytosis (AMT), magnetically guided nanoparticles, viral vectors, and exosomes, have demonstrated promising outcomes, significant limitations still need to be addressed. RMT-based nanoparticles achieve high specificity but often suffer from limited payload capacities and receptor saturation issues. AMT offers versatility but lacks specificity, which can potentially lead to off-target accumulation and cytotoxicity. Magnetically guided systems demonstrate improved precision but require external equipment and encounter challenges in scaling up to human trials. Viral vectors exhibit efficient transduction but carry risks of immunogenicity, and exosomes, while biocompatible, present challenges in terms of production scalability. In our view, these limitations emphasize that no single strategy will provide a universal solution; instead, progress will depend on carefully designed combinations of approaches and the rational tailoring of nanocarriers to specific therapeutic contexts.

A significant limitation in the clinical translation of BBB-crossing nanobiomaterials is the scarcity of robust clinical data. Translational medicine faces substantial barriers, primarily due to the inadequate characterization of safety and efficacy profiles. A critical step forward involves implementing well-designed preclinical studies that follow a Quality by Design (QbD) approach, ensuring a rigorous evaluation of critical attributes before clinical trials. From our perspective, prioritizing standardized protocols for preclinical assessment and harmonizing them across laboratories would accelerate the field by making data more comparable and reproducible, which is currently one of the main obstacles to translation.

Before progressing to clinical application, CNS-targeted nanomedicines must undergo rigorous safety and regulatory assessments. To facilitate this essential stage, Table 6 provides an organized overview of critical pre-in vivo toxicological evaluations, including hemocompatibility, neurotoxicity, and long-term accumulation, along with specific regulatory references and recommended methodologies. This structured approach ensures comprehensive characterization and regulatory compliance for NPs designed to cross the BBB. We believe that coupling these evaluations with early dialogue with regulatory agencies (FDA, EMA) could shorten the time to translation by anticipating safety concerns at the design stage, rather than only during late-stage validation.

Hemocompatibility assessments (hemolysis < 5%, minimal platelet activation, and low complement activation) ensure intravenous safety, mitigating risks of thrombotic and immune-mediated adverse events [178]. For neurotoxicity, CNS-targeted NPs should demonstrate no significant behavioral impairments or histopathological abnormalities at therapeutic doses, aligning with safety pharmacology guidelines [179,194]. Moreover, long-term accumulation studies must confirm that NPs are cleared from the CNS over time, ideally exhibiting >90% elimination within weeks post-administration, thereby minimizing delayed neuroinflammation or chronic toxicity [202].

Regulatory bodies (FDA and EMA) mandate comprehensive biodistribution and pharmacokinetic profiling, necessitating quantitative in vivo analyses using labeled nanomaterials to map NP fate, organ retention, and BBB crossing efficiency [190]. Likewise, immunotoxicity evaluations must preclude excessive immune activation or complement-mediated reactions (CARPA), verifying that anti-NP antibodies or cytokine storms are absent or manageable [195]. In our opinion, the inclusion of patient-derived models and advanced imaging tools in these studies could provide earlier and more human-relevant safety signals, bridging the gap between preclinical and clinical stages.

Finally, achieving manufacturing reproducibility during scale-up is pivotal. Establishing and maintaining Critical Quality Attributes (CQAs), such as particle size, polydispersity (PDI), zeta potential, and drug loading, across batches ensures consistent therapeutic outcomes and regulatory compliance [203]. Nanomedicine developers should proactively define and validate manufacturing processes to control batch-to-batch variability, leveraging GMP practices and possibly advanced techniques like microfluidics to maintain quality during scale transitions. From our perspective, the next decade will require stronger integration between academia and industry to develop scalable, cost-effective platforms that not only meet regulatory standards but also facilitate equitable access to CNS nanomedicines worldwide. Collectively, these data-driven recommendations form a robust foundation for safely advancing CNS-targeted NPs from preclinical validation towards clinical implementation.

## 6. Conclusions

This comprehensive guidance establishes a practical and rigorous framework for rationally designing and validating nanobiotechnological systems capable of effectively traversing the BBB. The summarized tables delineate key parameters for optimal NP physicochemical properties, targeting mechanisms, and validated experimental models, guiding researchers through informed and methodical design decisions. By integrating detailed considerations of NP size, charge, composition, as well as regulatory and toxicological standards, this guide enables the precision engineering of nanocarriers that demonstrate improved CNS targeting, therapeutic efficacy, and safety profiles.

Despite these advances, significant translational hurdles remain, particularly in terms of scale-up reproducibility and rigorous safety validation. Future research should focus on systematically addressing these practical challenges, employing the outlined toxicological assays, immunogenicity assessments, and pharmacokinetic evaluations. Proactively tackling these aspects will accelerate the clinical translation of innovative therapies for neurological disorders.

In summary, our contribution is a design-first synthesis that (i) defines numerical BBB design windows, (ii) provides a human-relevant model guide with decision points, and (iii) embeds a regulatory/toxicology checklist to de-risk translation, a scope not combined in prior reviews.

## Figures and Tables

**Table 1:** Summary of major transport mechanisms across the BBB.

Mechanism	Molecule Type	Size Limit	Transport Rate/Efficiency	Key Examples	References
Passive diffusion	Small, lipophilic	<400–500 Da	~2–6% small molecules	O_2_, CO_2_, ethanol, nicotine	[2,34]
Paracellular transport	Small ions, water	~1–2 nm pore size	10^−7^–10^−8^ cm/s (e.g., sucrose ~340 Da)	Water, sucrose	[4,27,28]
Carrier-mediated transport (CMT)	Polar nutrients	Variable (nutrient-specific)	GLUT1: ~0.5 μmol/g/min (glucose uptake)	Glucose (GLUT1), leucine (LAT1)	[33]
Receptor-mediated transcytosis (RMT)	Specific peptides/proteins	Extensive (kDa range)	~0.1–2% injected dose (antibody conjugates)	TfR, LDLR, insulin receptor	[37]
Adsorptive-mediated transcytosis (AMT)	Cationic/amphipathic macromolecules	Large (proteins, NPs)	~10–20-fold increase (cationic albumin)	Cationic albumin, TAT peptide	[39]

GLUT1: glucose transporter 1; LAT1: large neutral amino acid transporter 1; TfR: transferrin receptor; LDLR: low-density lipoprotein receptor; TAT: trans-activator of transcription.

**Table 2:** Experimental models for NPs delivery across the BBB.

Model Type	Specific Description	Preparation/Sourcing	Key Characteristics	Advantages	Limitations	Recommended Use Cases	References
In Vitro	Immortalized Cell Monolayer (hCMEC/D3), human brain endothelial cell line grown as a monolayer on inserts	Human brain endothelial cells immortalized (hTERT, SV40), cultured on collagen-coated Transwell inserts.	Retains human BBB markers but forms a leaky barrier (TEER ~30–100 Ω·cm^2^), low tight junction expression, and reduced efflux transporter activity (e.g., P-gp) compared to in vivo.	Simple, robust, high-throughput human model for rapid screening, drug uptake, and toxicity assays; expresses key BBB transporters and enzymes.	Very low barrier tightness (low TEER, high permeability); incomplete tight junctions; limited transporter expression; lacks astrocytes, pericytes, and flow; poor in vivo predictor.	High-throughput initial screening for NP BBB penetration, cytotoxicity, and human-specific transport; limited permeability precision requires validation in more stringent models.	[43,44,45,46]
In Vitro	Primary Endothelial Co-culture, e.g., primary rodent or porcine brain endothelial cells with astrocytes/pericytes	Freshly isolated brain endothelial cells seeded on permeable inserts, co-cultured with astrocytes (direct/indirect) to induce BBB phenotype, with optional inclusion of pericytes or primary human cells.	It develops tight junctions and exhibits low permeability, similar to in vivo conditions. Glial co-culture elevates TEER (porcine > rat > mouse). Expresses major BBB transporters (polarized P-gp, BCRP) and influx receptors (transferrin). Mimics in vivo drug exclusion (low paracellular flux).	Physiologically relevant model (high TEER, correct tight junctions, and native transporter activity); supports endothelial–glial interactions; BBB regulation; gold standard for permeability assays matching in vivo results.	Labor-intensive, low yield, requiring fresh tissue and specialized isolation; high batch variability and a short lifespan. Animal cells differ from human BBB; primary human cells are scarce and rapidly lose BBB properties (TEER ~40–50 Ω·cm^2^).	Mechanistic studies under near-physiological conditions; moderate-throughput screening with a tighter barrier than cell lines; validates simpler models; species selection based on goals; human model confirmation recommended.	[47,48,49]
In Vitro	hiPSC-Derived BBB-on-Chip—human iPSC-derived endothelial cells with astrocytes/pericytes in a microfluidic device.	hiPSCs differentiated to endothelial-like cells (Wnt/RA), cultured on Transwell or chip with astrocytes/pericytes. Shear flow and stimuli (e.g., hypoxia) improve maturation.	Human-like BBB phenotype: correct tight junctions (claudin-5, ZO-1, occludin); high TEER (~1000–5000 Ω·cm^2^); relevant transporter/receptor expression, efflux pumps (P-gp, BCRP; sometimes reduced activity); supports dynamic modulation.	An entirely human BBB model avoids species differences, maintains high barrier integrity (TEER > 20,000 Ω·cm^2^, >2 weeks), supports patient-derived iPSCs, and enables mechanistic and permeability studies.	Reflects systemic influences (metabolism, protein binding, and immune clearance). Measures real brain uptake and therapeutic effects. Genetically tractable. Cost-effective and accessible.	Human BBB model is ideal for preclinical NP testing, transport mechanism studies, and validating human-specific transporter targeting; suited for focused, low-throughput studies.	[40,41]
In Vivo	Mouse Model—rodent in vivo BBB (adult mice, healthy or disease models)	Brain uptake is assessed via post-mortem analysis, imaging (MRI/PET), or genetic models.	Complete neurovascular unit with tight junctions, high TEER (~1000–6000 Ω·cm^2^), dynamic blood flow, active efflux, and realistic NP biodistribution. Note: Higher P-gp expression in mice than in humans.	Captures systemic factors influencing NP delivery. Directly measures brain uptake and efficacy. Genetically modifiable (e.g., Mdr1a^−/−^). Cost-effective; suitable for disease modeling.	Species differences may underestimate human BBB uptake; small size complicates surgery and sampling; limited blood volume and rapid metabolism affect NP circulation; low throughput requires ethical approval; results require confirmation in other models.	In vivo proof-of-concept to confirm NP BBB crossing and payload delivery. Mechanistic analyses and preclinical efficacy testing. Intermediate validation step: positive mouse results typically require follow-up in NHPs for human translation.	[42,50,51]
In Vivo	Non-Human Primate Model—rhesus or cynomolgus macaque BBB in vivo.	NPs are administered intravenously (often under anesthesia), with brain uptake monitored by MRI, PET, or post-mortem analysis. Allows for repeated blood/CSF sampling. Small sample sizes (N ≤ 4–6) due to cost and ethical constraints.	Most similar to human BBB in structure and function. Monkeys share transporter profiles nearly identical to those of humans (96% amino acid identity in P-gp). Comparable brain anatomy, capillary tight junctions, and pericyte coverage. Enables NP testing in a human-like brain.	Highly predictive of human BBB outcomes. Supports clinical imaging (PET, SPECT) for detailed in vivo tracking of nanoparticles. Captures physiological BBB modulators, ensuring translational relevance. Essential for safety/toxicology evaluations required by regulatory agencies.	High cost, ethical, and logistical complexity limit throughput and statistical power. Requires specialized facilities and veterinary expertise. Minor physiological differences from humans exist. Handling stress and anesthesia may affect BBB properties. Genetic manipulation is impractical.	Late-stage validation confirms NP BBB crossing, safety, and pharmacokinetics before human trials. The final translational step uses primate models with human-like BBB and metabolism. This step is not for screening but informs critical go/no-go decisions.	[52,53]
In Vivo	Zebrafish Larval Model—zebrafish embryo/larva with developing BBB	Transparent zebrafish embryos (~3 dpf) with functional BBB. NPs are administered by microinjection or water exposure. Fluorescent transgenic lines visualize NP crossing of the BBB in vivo.	Zebrafish BBB with tight junctions and conserved transporters form by 3–4 dpf, selectively restricting molecules similarly to mammals. Key regulators (e.g., Mfsd2a) share analogous functions. Enables live tracking of NP BBB crossing.	The high-throughput, low-cost in vivo model enables parallel testing, real-time imaging, and genetic manipulation, with fewer ethical constraints.	The non-mammalian model exhibits differences in BBB maturity, immunity, and pharmacokinetics, resulting in limited predictive value, which necessitates mammalian validation and consideration of injection variability for certain NPs.	Rapid, early-stage in vivo screening of NP brain uptake and toxicity. Ideal for visualizing NP–BBB interactions. Helpful intermediate step before rodent studies; positive hits require mammalian validation.	[54,55,56]

**Table 3:** Comparative summary of nanoparticle materials for brain drug delivery: advantages, limitations, and references.

Material Type (Examples)	Advantages	Limitations	References
Lipid-based NPs (liposomes, solid lipid NPs)	High biocompatibility: Mimic cell membranes; low toxicity. Effective BBB crossing: Ligand functionalization significantly improves brain uptake (~5× vs. untargeted). Efficient genetic cargo delivery: Excellent mRNA transfection; minimal off-target accumulation.	Limited BBB penetration: PEGylated liposomes show poor brain uptake unless ligand-functionalized. Stability issues: Drug leakage and instability (e.g., unsaturated lipids release payload quickly). Require stabilization (e.g., PEGylation, cholesterol) to prevent aggregation and fusion. Rapid RES clearance: Unmodified liposomes are quickly opsonized and removed by macrophages (liver/spleen). PEGylation increases circulation but may reduce cellular uptake.	[90,92,93]
Polymeric NPs (PLGA, PLA, etc.)	Biodegradable and biocompatible: FDA-approved PLA/PLGA minimizes toxicity. Tunable release: Polymer matrices allow controlled drug delivery. Surface versatility: Easy PEGylation or ligand attachment for BBB targeting (e.g., LDLR-targeted PLGA: ~4% vs. ~1% untargeted). Customizable shape (spheres, rods) optimizes biodistribution.	Incomplete biodegradation: High-molecular-weight or non-degradable polymers may accumulate. Potential cytotoxicity: Cationic/high-generation polymers risk cell and BBB damage; safety optimization is challenging. Limited BBB crossing: Unmodified polymeric NPs require PEGylation or ligands (e.g., surfactants, ApoE) for effective brain uptake.	[94,95,96]
Dendrimers (poly(propylenimine) PPI, PAMAM)	Multivalent BBB crossing: Highly branched polymers utilize both adsorptive (cationic surfaces) and receptor-mediated (ligand-attached) transcytosis mechanisms. Enhanced targeted delivery: Transferrin/lactoferrin-PPI dendrimers achieved a >6-fold higher gene delivery efficiency; Angiopep-2-PPI dendrimers improved paclitaxel uptake in glioma cells. CNS therapeutic efficacy: Specialized dendrimers (e.g., maltose–histidine G4 PPI) crossed the BBB and preserved cognition in Alzheimer’s models; versatile conjugation of drugs/genes for targeted therapy.	Intrinsic cytotoxicity: Higher-generation cationic dendrimers disrupt cell membranes; require surface modifications (e.g., sugars, PEG) to reduce toxicity. Non-biodegradable: Many dendrimers (PPI, PAMAM) persist in the liver/spleen, causing long-term accumulation. Developing degradable dendrimers is challenging. Immunogenicity/biocompatibility: Can induce complement activation or oxidative stress; repeated dosing demands extensive surface engineering for safety.	[97,98]
Protein-based NPs (ferritin, albumin, virus-like particles)	Natural transport pathways: Ferritin/transferrin-based NPs use transferrin receptor transcytosis for inherent BBB targeting (e.g., human H-ferritin delivers antibodies into brain tumors). High biocompatibility: Endogenous/recombinant protein NPs (albumin, VLPs) are biodegradable, enzymatically degradable, and non-toxic. Easy surface modification: Can attach peptides or antibodies (e.g., RVG29, angiopep-2, transferrin) for enhanced specificity; naturally multivalent ligands (e.g., VLPs) increase targeting density.	Immunogenicity risk: Protein carriers can trigger immune responses, especially with repeated doses or non-human sources, requiring stealth modifications (PEGylation/humanization). Stability issues: Proteins can easily aggregate or denature during formulation and storage, reducing their efficacy; therefore, cold-chain storage or the use of lyoprotectants is often necessary. Limited drug loading: Protein nanocages have a restricted interior space, limiting the size and amount of the payload, which may mean higher doses are required for a therapeutic effect.	[86,99]
Inorganic NPs (gold, iron oxide, silica)	Stable cores: Structurally robust inorganic NPs (metal/mineral) that are precisely tunable (1–100 nm), optimizing BBB crossing (e.g., ultrasmall gold NPs < 10 nm transit intact BBB). Multifunctional theranostics: Intrinsic imaging/therapy capabilities (gold: CT/photothermal; iron oxide: MRI; silica: drug carrier/imaging). Easy functionalization: Surfaces (gold/silica) readily attach ligands/coatings (thiols, silanes, polymers), enhancing targeting (~50 nm gold NPs: ~5× higher brain uptake) and colloidal stability.	Non-degradable: Inorganic cores (gold, silica, metal oxides) persist in organs (such as the liver, spleen, and brain), causing long-term accumulation and potential chronic toxicity. Potential delayed toxicity: Oxidative stress or inflammation from NP degradation, surface exposure, or ion release (e.g., iron oxide > 50 nm: oxidative stress; gold NPs < 5 nm: cellular disruption). Limited BBB transit: Large, unmodified inorganic NPs exhibit poor brain uptake; achieving therapeutic levels requires ultrasmall size (<10 nm), targeted delivery, or BBB disruption techniques (e.g., magnetic guidance, ultrasound).	[100,101]

**Table 4:** Comparative data on NPs crossing the BBB.

Class	NPs (Formulation and Surface)	Size	Zeta	Brain Uptake	t_1_/_2_	Therapeutic Efficacy	Model	References
Lipid-based	Liposome—PEGylated (untargeted)	~90 nm	Negative	0.023% ID/g (4 h post-IV)	Short (less than targeted)	N/A (no CNS therapy tested; baseline delivery)	Healthy mice (C57BL/6)	[102]
Lipid-based	Liposome–scFv antibody-targeted (BBB receptor-specific)	~90 nm	Negative	0.24% ID/g (4 h; ~10× over untargeted)	Longer circulation vs. Control	Improved brain drug levels (2-PAM); distribution study (no disease model)	Healthy mice	[102]
Lipid-based	Liposome—TAT peptide-functionalized	~100 nm (est.)	Cationic (+)	~0.1% ID/g (1 h; ~background level)	NA	N/A (no improved uptake; no efficacy)	Healthy mice	[103,104,105]
Polymeric	PLGA NP—Poloxamer 188-coated (MTX + PTX combo)	133 nm and 221 nm	−29 mV/−18 mV nature.com	17.2% ID/g (48 h post-IV)	Detected in the brain up to 48 h	↓ Tumor volume, Ki-67; improved survival vs. control	Rat glioma (C6 orthotopic)	[106]
Polymeric	PLGA NP—unmodified (PEG-PLGA)	~100 nm (typical)	≈−15 mV (typical)	<1% ID/g (generally low)	Hours (moderate)	N/A (minimal BBB penetration)	Healthy rodents (general)	[107]
Polymeric	PAMAM Dendrimer—G4 (OH-terminated)	4.3 nm	~0 mV	1.9 ± 0.3 μg/g in tumor (24 h)	Rapid renal clearance	N/A (carrier targeted to microglia/Mϕ)	Rat 9L gliosarcoma/GL261 GBM	[108]
Polymeric	PAMAM Dendrimer—G6 (OH-terminated)	6.7 nm	~0 mV	17.6 ± 4.5 μg/g in tumor (24 h)	Extended (slower clearance)	N/A (selective TAM uptake; immunotherapy vehicle)	Mouse GL261 GBM	[108]
Protein-based	H-Ferritin nanocage (human heavy-chain)	~12 nm	−(native)	Effective BBB penetration; slow clearance in brain	Long (persistent in brain)	N/A (proposed CNS drug carrier; no drug loaded)	Healthy mice	[109]
Protein-based	Virus-Like Particle (JC polyomavirus VLP)	~40 nm	NA	~0% ID/g (negligible brain uptake after IV)	NA	N/A (gene vector; no therapeutic cargo in study)	Healthy mice (IV vs. carotid)	[110,111]
Inorganic	Gold NP—PEGylated (no targeting)	~15 nm (core)	~0 mV (PEG-coated)	0.04% ID/g (baseline)	~2.3 h	N/A (used as BBB photomodulation agent)	Healthy mice	[112]
Inorganic	Gold NP—anti-JAM-A antibody (BV11) coated	~15 nm (core)	~0 mV	0.13% ID/g (baseline; ~3× PEG-NP)	~0.17 h (≈10 min)	N/A (facilitates laser-induced BBB opening)	Healthy mice	[112]
PPI dendrimer	Lactoferrin- and lactoferricin-conjugated PPI dendrimers (Gen.3) complexed with TNF-α plasmid DNA (dual-targeted dendriplex)	~150 nm (polyplex)	Positive (cationic) at all DNA ratios.	Yes, targeted delivery to tumors; higher tumor uptake vs. non-targeted (lower liver uptake).	NA	Complete tumor regression in 60% of A431 tumors and 50% of B16-F10 tumors (one month) after IV treatment; well-tolerated.	Tumor-bearing mice (A431 xenograft and B16-F10 melanoma).	[113]
PPI dendrimer	Histidine–maltose shell PPI dendrimer (G4HisMal) (glyco-modified dendrimer, no drug; neuroprotective agent)	~6 nm (monomer)	~Neutral (sugar-modified)	Yes, enhanced BBB penetration (intranasal delivery gave 40% higher brain level vs. non-histidine control)	Not reported	Memory rescue: Treated APP/PS1 Alzheimer’s mice showed significantly improved memory vs. controls and preserved synaptic markers	APP/PS1 transgenic AD mice (±Aβ in vitro)	[114]

NA: Not assessed.

**Table 5:** Comparison of nanotechnological strategies for BBB crossing, highlighting the delivery system, mechanism, NP type, model, brain uptake, and therapeutic effects.

Strategy and System	Target/Mechanism	NP or Vector	Model Used	Brain Uptake Metrics	Therapeutic Outcome	References
TfR-mediated (RMT), e.g., OX26 antibody NP.	Transferrin receptor on the BBB endothelium.	OX26-conjugated PEGylated liposome or gold NP.	Rat (in vivo)	~0.3% ID in brain vs. 0.03% for IgG (10-fold increase); parenchymal 0.23% ID/g with optimized affinity.	Enhanced brain drug levels; basis for enzyme therapy (ETV:IDS) yielding 50–76% substrate reduction in CNS.	[117,118,133]
T7-PLGA NPs	Transferrin receptor (TfR) targeting, T7 peptide (HAIYPRH) binds TfR on the BBB endothelium, triggering receptor-mediated transcytosis.	PLGA polymer NPs decorated with T7 peptide (often PEGylated; can carry drugs or genes).	Murine brain tumor models (orthotopic glioma) and healthy mice (distribution studies).	T7-functionalized NPs increased brain accumulation by ~6-fold, gene expression by 1.7-fold, and photosensitizer delivery to gliomas by ~6-fold versus untargeted controls.	T7-targeting improved outcomes: T7-liposomes (ZL006) reduced infarct volume and improved neurological recovery in stroke; T7-NPs enhanced tumor suppression and survival in glioma.	[154,155,156]
INSR-mediated (RMT), e.g., 29B4 antibody.	Insulin receptor (ubiquitous, BBB, and neurons).	Human insulin receptor mAb (29B4) on HSA NP.	Mouse (in vivo)	Qualitative crossing confirmed (therapeutic levels achieved); clinical fusion protein ~2–3% CSF: plasma ratio in patients (phase 1).	CNS enzyme delivery in Hunter syndrome (valanafusp alpha)—reduced CNS pathology; in rodents, INSR-NPs showed functional neuroprotection.	[119,120,157]
LRP1-mediated (RMT): Angiopep-2 peptide.	LRP1 on the endothelium (also in tumors).	Angiopep-2 decorated polymeric NP.	Mouse (in vivo); in vitro BBB models.	~2–4× higher brain uptake vs. non-targeted NP (biodistribution studies); transcytosis of Ang2-NPs observed in iPSC-derived human BBB model.	In patients, Ang2-NP delivering paclitaxel (ANG1005) showed tumor shrinkage; Ang2-polymersomes with carnosine reduced stroke infarct volume.	[121,122,158]
Angiopep-2 lipid–silica NPs	Angiopep-2 targets LRP1 receptors on the BBB and glioma cells, mediating transcytosis into the brain.	Lipid-coated mesoporous silica NPs loaded with paclitaxel and functionalized with Angiopep-2.	Rat intracranial glioma model (C6 glioma-bearing rats; IV administration).	Angiopep-2 NPs enhanced paclitaxel brain delivery (~20.6% vs. ~10.6% targeting efficiency), doubling brain drug concentrations compared to untargeted controls.	Angiopep-2 targeting enhanced brain tumor therapy, prolonging survival and increasing tumor apoptosis compared to untargeted NPs.	[159]
Lactoferrin R-mediated (RMT)—Lf-NC.	Lactoferrin receptor (on BBB and glioma cells).	Lactoferrin-coated lipid nanocapsule (Lf-LNC).	Rat (PTZ epilepsy model).	Brain APO concentration ↑ (significant, e.g., 1.5-fold vs. uncoated); Lf coating improved BBB permeability.	Suppressed seizures: ~0.67 Racine score with Lf-LNC vs. ~3 (uncoated); reduced neuroinflammation.	[127,128,160]
Folate-mediated (RMT)—FA-NP.	Folate receptor-α (high in glioma, low BBB).	Folic acid-conjugated gold NP (or polymer NP).	Mouse glioma (orthotopic)	Tumor: brain ratio > 5:1 uptake in FR-positive tumor; minimal uptake in normal brain.	Enhanced GBM cell kill and imaging contrast; extended survival in folate-R expressing tumor models.	[131,132,161]
Adsorptive (AMT)—PepH3 peptide NP.	Electrostatic adsorptive uptake.	PepH3 (7-aa cationic) tagged vesicular NP.	Rat and human BBB cell culture; Mouse IV.	Endothelial uptake ↑ (~3–5× vs. no peptide); in vivo high brain localization, low off-target (radiotracer).	Delivered anti-Aβ single-domain antibody across the BBB in vitro; potential Alzheimer’s therapy shuttle (in vivo efficacy pending).	[135,136,137]
Adsorptive (AMT)—Chitosan NP.	Electrostatic (polycationic polymer)	Chitosan DNA NP (~260 nm)	Mouse (in vivo, i.p. injection).	Confirmed BBB crossing: GFP gene expressed in brain cells; brain transfection efficiency ~53% (FACS, vs. 27% with control vector).	Successfully expressed therapeutic gene (GFP) in brain parenchyma; proof-of-concept for gene therapy in brain tumors or neurodegeneration.	[139]
Magnetic Targeting—Liposomal SPION (LTF).	External static magnetic field (SMF) guides NP.	Temozolomide + ferucarbotran liposome (LTF).	Mouse glioma (GL261 in the brain).	The tumor NP concentration was ~2 times higher in the magnet (MRI-based) group; the magnet-guided group showed a *p* < 0.01 reduction in tumor volume by day 7.	Tumor growth suppressed; median survival ↑ vs. non-magnet (e.g., ~25 days to >31 days with magnet).	[142]
Magnetic nanocapsules	Magnetic targeting via an external field enhances the transcytosis of iron oxide nanocapsules across the BBB.	~100 nm silica-coated magnetic NPs (iron oxide core) with RF-triggered drug-release capability.	Healthy mice with intact BBBs were subjected to localized magnetic targeting post-IV injection.	Localized magnetic fields increased brain NP delivery by ~25–26-fold versus controls; ~30% of peak brain signal persisted at 48 h, while non-magnetized delivery remained near background levels.	Magnetic nanocapsules enabled non-invasive BBB crossing without acute toxicity; histology confirmed vessel integrity. Though therapeutic efficacy remains untested, they allow for on-demand drug release via radio-frequency heating.	[162,163]
Magnetic Targeting, Tween-SPION.	Magnetic field induces BBB transport.	20 nm Tween-80 coated SPIONs.	Rat (normal BBB, iv + EMF).	Crossed intact BBB under EMF; SPIONs detected in brain parenchyma (none without EMF).	No therapeutic payload (diagnostic); demonstrates non-invasive BBB crossing by physical force.	[142]
Viral Vector—Engineered AAV (CAP-Mac).	Capsid-mediated transcytosis (evolved tropism).	AAV.CAP-Mac (neurotropic AAV variant).	Non-human primates (marmoset, macaque).	~1.1–1.3% of all neurons transduced (green monkey) vs. <0.5% with AAV9; broad CNS distribution (11 of 11 regions positive).	Enabled IV gene delivery, e.g., widespread GCaMP expression for imagingnature.com; supports CNS gene therapy (potential for autism, Alzheimer’s).	[145]
Viral Vector—RVG-pseudotyped LV.	Viral glycoprotein-mediated entry.	Lentivirus coated with RVG peptide.	Mouse (in vivo).	Qualitative BBB crossing (RVG-LV detected in brain, unlike unmodified LV); transgene in neurons.	Partial motor function restoration in a neurodegenerative mouse model (using RVG-LV to deliver therapeutic gene).	[144,151]
TMV-VLPs	Size/shape EPR + ligand (albumin, etc.)	Tobacco Mosaic Virus nanorod + albumin coat	Mouse (brain tumor model)	Accumulated in brain tumor (MRI and NIR imaging); higher tumor: normal brain ratio than spherical NP.	Improved tumor imaging and delivery of photothermal therapy; significant tumor cell apoptosis in combination treatment.	[77,164]
CCMV VLPs	None (passive) Natural 28 nm protein cage (plant virus capsid) with no specific targeting; crosses BBB at low levels, possibly via adsorptive transcytosis.	Empty CCMV capsid as a drug nanocarrier.	Healthy mice (IV injection, no disruption of BBB).	Approximately 0.3% ID/g was detected in the brain at one h post-injection, decreasing to <0.01% by 24 h; brain distribution was comparable to other protein-based NPs.	No therapeutic payload was tested; safety studies showed no overt toxicity or immune response in mice.	[165]
Exosome—RVG-modified MSC exosomes	Endogenous vesicle uptake + neuron targeting	RVG-peptide engineered exosomes (MSC-derived)	Mouse Alzheimer’s model	Preferential localization to cortex/hippocampus; exosomal cargo (siRNA) in brain increased ~2× vs. free siRNA.	Restored memory function (exosome-treated mice performed significantly better in Morris water maze); reduced brain Aβ and inflammation.	[151,166,167]
Exosome—Cetuximab-Exo-Dox	Endogenous vesicle + tumor targeting (EGFR)	Exosomes loaded with doxorubicin + Cetuximab	Mouse glioblastoma model	Brain delivery of cetuximab ↑ (~2-fold) with exosomes vs. free Ab; doxorubicin brain concentration also higher (HPLC quantification).	Enhanced GBM growth inhibition and prolonged survival vs. free drug; exo combo therapy induced greater tumor cell apoptosis (histology).	[152]
MSC-derived exosomes	MSC-derived exosomes (~50–150 nm) cross the BBB via endocytosis, with enhanced uptake under inflammatory conditions.	MSC-derived EVs carrying therapeutic cargo (proteins/miRNA or drugs).	Rodent models of CNS injury (stroke, TBI) for therapy; healthy rats for biodistribution.	Baseline brain uptake was low (~0.03–0.04% ID/g) after IV administration; entry increased in neuroinflammatory models with preferential accumulation in injured regions.	MSC-exosomes reduced infarct volume by ~50%, improved neurological function in stroke, and attenuated neuroinflammation with cognitive recovery in TBI models.	[168,169,170,171,172,173,174]
Micromotor—NIR Janus nanomotor	Photothermal propulsion (active movement)	Gold–Janus NPs (NIR-responsive)	Mouse (in vivo experiment)	BBB penetration significantly improved under NIR (qualitative: increased dye leakage into brain); no crossing without NIR.	Facilitated brain delivery of a model drug (dye) with spatiotemporal control; concept validated for on-demand BBB opening.	[175]
Macrophage-mediated “Trojan Horse” delivery	Monocytes/macrophages naturally cross the BBB, delivering internalized drug-loaded NPs to inflamed or tumor sites.	Macrophages loaded ex vivo with drug-encapsulated NPs, then injected intravenously.	Mouse glioblastoma and neuroinflammation (e.g., Parkinson’s) models using macrophage adoptive transfer.	NP-loaded macrophages greatly enhanced brain tumor localization versus free NPs; intrathecal macrophage transfer achieved ~8.1% ID/g brain uptake, surpassing standard IV delivery.	Macrophage-mediated delivery improved glioma drug deposition, potentially limiting tumor growth, and increased GDNF levels with functional recovery in Parkinson’s, leveraging immune-cell homing to the CNS.	[176]
Microrobot—Magnetic spiral (platelet cloaked)	Magnetic rotation (swimming)	Helical nanorobot with Fe coating + platelet membrane	In vitro blood flow; proposed in vivo mouse	Propulsion sustained in blood-mimicking flow; able to navigate and marginate toward vessel walls. (BBB crossing has not yet been directly measured).	Demonstrated long circulation and targeting potential; aims to mechanically traverse the BBB and deliver drugs (studies in progress).	[177]

Quantitative brain uptake is given where available (percentage of injected dose in brain, %ID/g tissue, or fold-change vs. controls). Arrow notation: ↑ increase; ↓ decrease; ↔ no significant change; → “leads to/compared to” (or “to” when indicating a change in values). ID: injected dose; TfR: transferrin receptor; INSR: insulin receptor; LRP1: low-density lipoprotein receptor-related protein-1; FR: folate receptor; RMT: receptor-mediated transcytosis; AMT: adsorptive-mediated transcytosis; SPION: superparamagnetic iron oxide NP; EMF: electromagnetic field; AAV: adeno-associated virus; LV: lentivirus; VLP: virus-like particle; RVG: rabies virus glycoprotein peptide; MSC: mesenchymal stem cell; EGFR: epidermal growth factor receptor; NIR: near-infrared; GCaMP: calcium indicator gene; siRNA: small interfering RNA: MSC: Mesenchymal stem cell; EVs: extracellular vesicles; TBI: traumatic brain injury; CCMV-VLPs: Cowpea chlorotic mottle virus-like particles; RF: remotely heated; TMV: Tobacco mosaic virus-like particles.

**Table 6:** Toxicological parameters, regulatory requirements, and scale-up challenges for BBB-targeted nanomaterials.

Parameter	Recommended Studies/Assays	Minimal Data Required (Thresholds)	Key Considerations	References
Hemocompatibility (blood compatibility of IV nanomedicine)	In vitro blood tests: Hemolysis assay (human RBCs); complement activation (C3a, C5a, SC5b-9); platelet aggregation/coagulation (platelet markers, thrombin, aPTT, PT).	Hemolysis: % hemoglobin release; <5% considered low risk (non-hemolytic). (ISO 10993-4 standard). Ideally, <2% (negligible hemolysis). Complement: No abnormal complement consumption or excessive anaphylatoxin rise (C3a/C5a) compared to negative control (i.e., should not trigger significant CARPA). Platelets/Coagulation: No significant platelet aggregation or >10% change in clotting times relative to baseline. (No official numeric limit; ensure values remain within a normal range of variation.)	Use fresh human blood to avoid species-specific platelets and complement differences. Account for NP interference with assay readouts via proper controls. Follow ISO 10993-4 and ASTM F756 standards to ensure hemocompatibility (to prevent thrombosis, hemolysis, and infusion reactions). If complement activation occurs in vitro, evaluate the risk of infusion reaction in vivo and consider methods for complement inhibition.	[16,178,179,180,181,182,183,184,185]
Neurotoxicity (CNS behavioral and histopathological safety)	Safety pharmacology and neurotoxicity: Perform FOB or modified Irwin test (locomotion, reflexes, coordination, sensorimotor responses, convulsions). Include behavioral assays (open field, rotarod, cognition if needed). Assess CNS histopathology (brain, spinal cord) in subchronic/chronic studies for neuronal/glial damage, inflammation, or vacuolization.	Neurobehavioral outcomes: No significant adverse effects on motor activity, gait, reflexes, or behavior at therapeutic levels. Minor changes (<20% compared to the control), reversible, and dose-dependent effects are acceptable. Neuropathology: No significant CNS lesions (neuronal degeneration, gliosis, demyelination) exist beyond background; minimal changes are allowed only at doses above therapeutic exposure. Neurofunctional tests: Grip strength, rotarod, and maze performance impairment should remain minimal (<10–15%) at clinical doses, excluding sedation effects.	FDA S7A Guidance: Evaluate CNS effects (behavior, reflexes, coordination, temperature), especially for CNS-targeted NPs. Assess neuroinflammation (microglia/astrocytes: Iba1, GFAP). Include behavioral tests for chronic CNS exposure. Use recovery groups to check reversibility.	[179,186,187,188,189]
Long-term Accumulation (Brain retention and clearance)	Biodistribution studies: Labeled NPs (radioactive/fluorescent) track long-term brain and organ distribution. Multiple time point assessments (weeks to months) are conducted, and imaging methods (PET/MRI) are preferred for non-invasive monitoring. Chronic toxicity: Extended observation periods post-treatment should be included to evaluate CNS persistence and delayed neurotoxicity. Brain and CSF should be analyzed at intervals to assess clearance.	Brain retention half-life: Biodegradable NPs should clear significantly (>50% within weeks); non-biodegradable NPs must plateau without progressive accumulation. Residual brain burden: post-treatment brain levels should substantially decrease (<10% peak) within 1–3 months. Clearance pathways: Identify clearance routes (e.g., glymphatic). Rapid clearance (hours–days) is preferred; persistent presence (>6 months) needs justification.	NP biodegradability: Assess persistence of nonbiodegradable NPs (e.g., gold, silica) and potential chronic neurotoxicity. Biological fate (FDA): Evaluate NP distribution, accumulation, and clearance from the brain and organs. Brain clearance: Examine glymphatic and phagocytic pathways; test in healthy and impaired clearance models. Human translation: Use animal retention data to inform human risk; persistent retention may require clinical imaging or dose adjustments.	[165,187,190,191,192,193]
Biodistribution and Pharmacokinetics (PK) (Systemic and CNS distribution, drug exposure)	Animal ADME studies: Radiolabeled or tracer methods track the distribution of NPs and their payloads over time. They measure plasma pharmacokinetics (Cmax, T½, AUC, clearance) and tissue distribution (e.g., percentage of dose in brain vs. organs). Brain penetration metrics: Calculate brain: plasma ratios or brain targeting indices. Include CSF levels if relevant. PK modeling: Apply compartmental or PBPK models using NP properties to predict human PK and dosing.	Animal ADME studies: Radiolabeled/tracer methods measure the distribution of nanoparticles and their payloads in plasma, brain, and organs over time. Report plasma PK (C_max, T_½, AUC, clearance) and tissue distribution (% dose in brain vs. other organs). Brain penetration: Calculate brain: plasma ratios or targeting index; include CSF levels if relevant. PK modeling: Apply compartmental or PBPK models with NP parameters to predict human PK and dosing.	Plasma half-life (T_1/2_): Report half-life versus free drug; nanoformulations typically extend circulation time (~10× longer than expected). Brain uptake: Higher brain/plasma ratios (>0.1 generally, >1.0 if targeted) indicate improved CNS targeting compared to a free drug. Bioavailability/distribution: Quantify brain delivery fraction. Minimize systemic exposure intrathecally; characterize off-target accumulation intravenously. PK linearity: Confirm dose-proportional exposure; investigate significant non-linearities (e.g., saturation, aggregation).	[190,194,195,196]
Immunogenicity and immunotoxicity (Regulatory requirement)	In vitro assays: Cytokine release (IL-6, TNFα, IFNγ) in human PBMCs. Complement activation (C5a, SC5b-9; CARPA risk). Immune cell function (macrophage uptake/ROS, T-cell activation, dendritic cell maturation). Myelosuppression (bone marrow colony assays). In vivo immunotoxicity: Evaluate immune organ histopathology (spleen, lymph nodes) and blood leukocyte subsets. If immunotoxic signals appear, targeted studies should be conducted per ICH S8. Monitor anti-drug antibodies (ADAs), especially against proteins or PEG.	Cytokine induction: Minimal cytokine release (e.g., IL-6 <3× baseline). Use controls for comparison. Complement activation: Low complement activation (<50% positive control). High levels signal hypersensitivity risk. Immunogenic antibodies: Monitor anti-nanoparticle antibodies; incidence typically ≤20%. Significant anti-PEG IgM or clearance changes (ABC phenomenon) require attention. Immune cell counts: Maintain WBC subsets within ±30% of control. Investigate consistent suppression or activation (e.g., T-cell drop, eosinophilia).	Nanomedicine immunogenicity: Assess risks (patient, route, dose). Include NP-specific assays (complement, inflammasome, immune cells). Carrier vs. Payload: Identify carrier (anti-PEG) vs. payload reactions; mitigate significant responses (e.g., ABC phenomenon). Clinical monitoring: Monitor immune reactions (anaphylaxis, complement). Investigate mechanisms; justify and plan mitigation strategies.	[183,190,195,197,198]
Scale-up and reproducibility	Chemistry, Manufacturing, and Controls (CMC): Implement robust cGMP processes. Characterize NP CQAs: particle size (DLS, laser diffraction), ζ-potential, morphology (TEM), drug loading/encapsulation (HPLC, spectroscopy), purity, endotoxin (LAL), and sterility (injectables). Conduct stability studies (size, potency, aggregation over time). Scale-up validation: Using statistical quality control, ensure batch consistency (size, PDI, drug content/release) between pilot and production scales.	Particle size and PDI: Maintain consistent size (±10% target), low PDI (<0.3; ideally ≤0.2). Avoid aggregates (>1000 nm). Drug content/release: Drug content within 90–110% of label; consistent batch-to-batch release profiles. Other CQAs: Stable zeta potential, impurities within ICH Q3D, endotoxin below USP limits (<5 EU/kg). Reproducibility: Consistent CQAs batch-to-batch (%RSD < 5–10%). Scale-up should not affect critical attributes.	A QbD approach is recommended to control critical parameters, ensuring consistent NP quality at scale. Analytical methods require NP-specific validation, reporting particle size confirmed by orthogonal methods. Adjustments during scale-up (e.g., homogenization parameters) and documented comparability (FDA/EMA guidelines) are essential. Regulatory compliance requires cGMP manufacturing, aseptic processing (especially if >200 nm), comprehensive CQA testing, and detailed CMC documentation before clinical approval.	[190,199,200,201]

ADAs: Anti-Drug Antibodies; ADME: Absorption, Distribution, Metabolism, Excretion; AMT: Adsorptive-Mediated Transcytosis; AUC: Area Under Curve; ASTM: American Society for Testing and Materials; BBB: Blood–Brain Barrier; CARPA: Complement Activation-Related Pseudoallergy; CMC: Chemistry, Manufacturing, and Controls; CNS: Central Nervous System; CQAs: Critical Quality Attributes; CSF: Cerebrospinal Fluid; DLS: Dynamic Light Scattering; EMA: European Medicines Agency; FDA: Food and Drug Administration; FOB: Functional Observational Battery; GFAP: Glial Fibrillary Acidic Protein; HPLC: High-Performance Liquid Chromatography; ICH: International Council for Harmonisation; IL-6: Interleukin-6; IV: Intravenous; LAL: Limulus Amebocyte Lysate; MRI: Magnetic Resonance Imaging; NOAEL: No Observed Adverse Effect Level; OECD: Organisation for Economic Co-operation and Development; PBMCs: Peripheral Blood Mononuclear Cells; PBPK: Physiologically Based Pharmacokinetic; PDI: Polydispersity Index; PEG: Polyethylene Glycol; PET: Positron Emission Tomography; PK: Pharmacokinetics; PT: Prothrombin Time; QbD: Quality by Design; RBCs: Red Blood Cells; RES: Reticuloendothelial System; ROS: Reactive Oxygen Species; TEM: Transmission Electron Microscopy; TNFα: Tumor Necrosis Factor-alpha; WBC: White Blood Cell; ζ-potential: Zeta Potential. **Notes:** When specific quantitative criteria are not established in guidelines, the values above are based on standard industry practices or literature and should be interpreted as general guidance, not fixed regulatory limits.

## Abbreviations

The following abbreviations are used in this manuscript:

BBB	Blood–Brain Barrier
NP	Nanoparticle
CNS	Central Nervous System
RMT	Receptor-Mediated Transcytosis
AMT	Adsorptive-Mediated Transcytosis
TfR	Transferrin Receptor
LDLR	Low-Density Lipoprotein Receptor
LRP-1	Low-Density Lipoprotein Receptor-Related Protein 1
GLUT1	Glucose Transporter 1
LAT1	Large Neutral Amino Acid Transporter 1
TAT	Trans-Activator of Transcription
hCMEC/D3	Human Cerebral Microvascular Endothelial Cell Line/D3
hiPSC	Human Induced Pluripotent Stem Cell
SPION	Superparamagnetic Iron Oxide Nanoparticle
GBM	Glioblastoma Multiforme
TEER	Transendothelial Electrical Resistance
PEG	Polyethylene Glycol
RES	Reticuloendothelial System
VLP	Virus-Like Particle
AuNPs	Gold Nanoparticles
APO	Apocynin
AAV	Adeno-Associated Virus
RVG	Rabies Virus Glycoprotein
MSC	Mesenchymal Stem Cell
CPP	Cell-Penetrating Peptide
NHP	Non-Human Primate
CSF	Cerebrospinal Fluid
TEM	Transmission Electron Microscopy
MRI	Magnetic Resonance Imaging
PET	Positron Emission Tomography
EMA	European Medicines Agency
FDA	Food and Drug Administration
GMP	Good Manufacturing Practice
CQAs	Critical Quality Attributes
CARPA	Complement Activation-Related Pseudoallergy
PK	Pharmacokinetics
ADME	Absorption, Distribution, Metabolism, and Excretion
PBPK	Physiologically Based Pharmacokinetic
ISO	International Organization for Standardization
ASTM	American Society for Testing and Materials
ICH	International Council for Harmonisation
LAL	Limulus Amebocyte Lysate
CMC	Chemistry, Manufacturing and Controls
PDI	Polydispersity Index
ABC	Accelerated Blood Clearance
